# The *Angelica dahurica*: A Review of Traditional Uses, Phytochemistry and Pharmacology

**DOI:** 10.3389/fphar.2022.896637

**Published:** 2022-07-01

**Authors:** Hui Zhao, Ya-Long Feng, Ming Wang, Jing-Jing Wang, Tian Liu, Jun Yu

**Affiliations:** ^1^ Clinical Experimental Center, Xi’an International Medical Center Hospital, Xi’an, China; ^2^ Xi’an Engineering Technology Research Center for Cardiovascular Active Peptides, Xi’an, China; ^3^ School of Chemistry and Chemical Engineering, Xianyang Normal University, Xianyang, China; ^4^ College of Food Science and Engineering, Northwest University, Xi’an, China; ^5^ Biomedicine Key Laboratory of Shaanxi Province, College of Life Science, Northwest University, Xi’an, China

**Keywords:** *Angelica dahurica*, coumarins, imperatorin, anti-inflammation, anti-tumor, review

## Abstract

*Angelica dahurica* (*A. dahurica*) root is a famous edible medicinal herb that has been used in China for thousands of years. To date, more than 300 chemical constituents have been discovered from *A. dahurica*. Among these ingredients, coumarins and volatile oils are the major active compounds. Moreover, a few other compounds have also been isolated from the root of *A. dahurica*, such as alkaloids, phenols, sterols, benzofurans, polyacetylenes and polysaccharides. Modern pharmacological studies demonstrated that the root of *A. dahurica* and its active components displayed various bioactivities such as anti-inflammation, anti-tumor, anti-oxidation, analgesic activity, antiviral and anti-microbial effects, effects on the cardiovascular system, neuroprotective function, hepatoprotective activity, effects on skin diseases and so on. Based on these studies, this review focused on the research publications of *A. dahurica* and aimed to summarize the advances in the traditional uses, phytochemistry and pharmacology which will provide reference for the further studies and applications of *A. dahurica*.

## 1 Introduction

*Angelica dahurica* (Hoffm.) Benth. & Hook.f. ex Franch. & Sav., belonging to Apiaceae family, exerts dual functions as medicine and food, which is pervasively distributed in eastern, northern and southeastern Asia. As a well-known traditional Chinese medicine (TCM), the root of *A. dahurica* (Chinese name:白芷) has been commonly used either alone or in combination with other herbal medicines to treat cold fever, headache, toothache, cold-damp pain and some skin diseases in China for centuries (Lee B.W. et al., 2020). Many classic formulas containing *A. dahurica* root have been widely used in clinic and have made important contributions to the health of people in China and other traditional medicinal systems in Asia. For example, the combination of *A. dahurica* root with *Atractylodes lancea* (Chinese name:苍术) could significantly enhance the effect of eliminating dampness, thus be used in treating arthrodynia. The combination of *A. dahurica* root with *Xanthium sibiricum* (Chinese name:苍耳子) has been commonly used for the treatment of rhinitis and nasosinusitis. In folk, *A. dahurica* root is often used as to make tea and health-care product, which is beneficial to treat cold-damp pain and rhinitis, and nourish blood. However, it is noteworthy that the vast and irrational use of *A. dahurica* root could lead to spasm and paralysis, and pregnant women and those with yin deficiency and blood heat should not use it.

**FIGURE 1:**
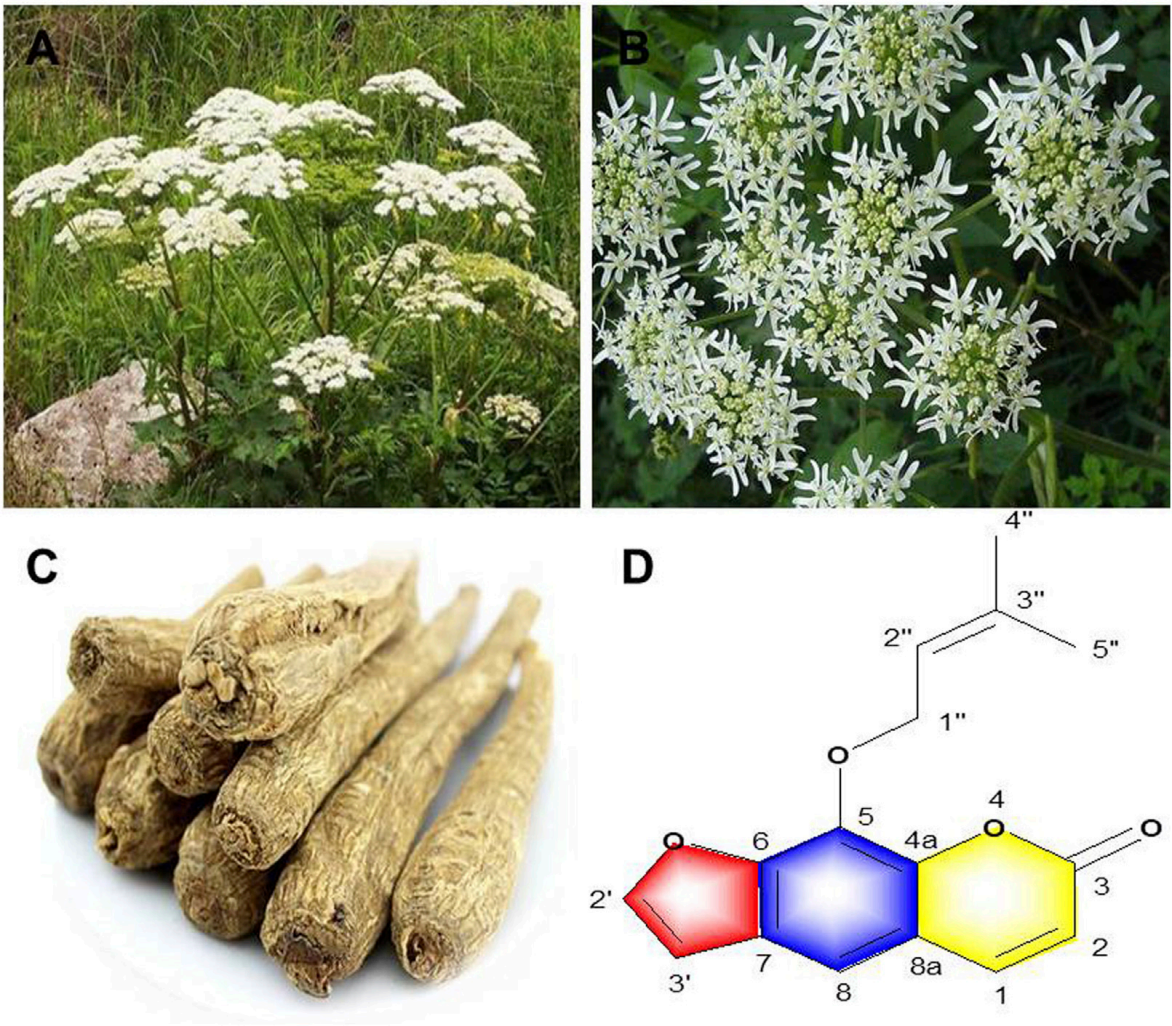
*A. dahurica*: **(A)** aerial parts **(B)** flowers **(C)** roots **(D)** chemical structure of imperatorin.

In the past few decades, *A. dahurica* root has attracted widespread attention as an important herbal medicine. Significant progress on isolation and identification of active constituent in *A. dahurica* has been made in relevant researches. Numerous studies have demonstrated that *A. dahurica* contains a broad spectrum of phytochemical constituents. The main chemical components of *A. dahurica* include coumarins and volatile oils, which are regarded as the representative constituents with putative bioactivities (Lee B.W. et al., 2020; Wu et al., 2016). There are more than 150 coumarins have been identified from *A. dahurica*, including simple coumarins, furanocoumarins, coumarins glycosides, other coumarins and coumarin derivatives. Among them, furanocoumarin imperatorin (22, IMP, Figure 1) is the most representative coumarin in *A. dahurica* root (Deng et al., 2020). The volatile oils isolated from *A. dahurica* mainly include terpenes, aromatics, alcohols, aldehydes, ketones, acids, esters and alkanes. Extensive studies indicated that *A. dahurica* exhibits a broad range of bioactivities, which can be attributed to the presence of multiple active components. Some of these bioactivities are consistent with the traditional uses of *A. dahurica* root, such as analgesic activity and effects on skin diseases. In addition, *A. dahurica* also exerts specific effects, related to anti-diabetic (Han et al., 2018), lowering blood lipids (Lu et al., 2016), improving immunity (Wang et al., 2021), anti-ulcer (Hu et al., 2021) and cosmetic effects (Cho et al., 2006).

Although *A. dahurica* has been widely studied on its chemical constituents and bioactivities, there is no comprehensive review about this edible medicinal herb. Therefore, the present article provides a systematic overview of *A. dahurica* covering its botany, traditional uses, phytochemistry, pharmacology, pharmacokinetics, quality control and safety. It is anticipated that this review will provide a new insight for the further study on the chemical constituents and bioactivities of *A. dahurica*.

## 2 Botany

*A. dahurica* is a member of Apiaceae family and is commonly distributed in eastern, northern and southeastern Asia (https://www.gbif.org). Wild *A. dahurica* often grows in forests, forest margins, streams, shrubs and valleys. Nowadays, *A. dahurica* is cultivated in many areas, and its roots are collected for medicinal purposes. As a perennial herb, *A. dahurica* grows to the height of 1–2.5 m **(** Figure 1 **)**. The root of *A. dahurica* is cylindrical with branches and its *epidermis* is tawny to brown with a strong smell. The stem of *A. dahurica* is hollow and 2–5 cm in diameter with the color of purple. The leaves are often ovate or triangular, with petioles up to 15 cm long. The flowers are compound umbels that are 10–30 cm in diameter with rough hairs in peduncles, rays and flower stalks. There are approximately 18–40 rays in *A. dahurica* and even as many as 70 in the center. *A. dahurica* fruits are round to ovoid with the color of yellowish-brown. The flowering phase ranges from July to August, and the mature fruit stage is typically from August to September (Flora of China Editorial Committee, 2006).

## 3 Traditional Uses

The root of *A. dahurica* has a long history of use and is characterized by pungent in taste and warm in nature. It has been widely used in TCM with excellent therapeutic effects for the treatment of cold, headache, forehead pain, epistaxis, nasosinusitis, toothache, abnormal leucorrhea in women and sore. An oral dosage of 3–10 g of *A. dahurica* has been recommended in the 2020 edition of Chinese pharmacopoeia. Moreover, the external use of *A. dahurica* root can treat boils, carbuncles, sores and painful swellings (Chinese Pharmacopoeia Commission, 2020). Dating back more than 1700 years of history, *A. dahurica* root was first documented in *“Shen Nong Ben Cao Jing”* (神农本草经) (Dong Han Dynasty, 25–220 A.D.), which is the earliest classic on TCM. Later, it was listed in many other well-known works on Chinese herb, including *“Ming Yi Bie Lu”* (名医别录) (Wei and Jin Dynasty, 220–420 A.D.), *“Yao Xing Lun”* (药性论) (Tang Dynasty, 618–907 A.D.), *“Ri Hua Zi Ben Cao”* (日华子本草) (Song Dynasty, 960–1279 A.D.), *“Dian Nan Ben Cao”* (滇南本草) (Ming Dynasty, 1,368–1644 A.D.) and *“Ben Cao Gang Mu”* (本草纲目) (Ming Dynasty, 1,368–1644 A.D.). The traditional uses of *A. dahurica* in ancient books of different dynasties are listed in Table 1. Similarly, the root of *A. dahurica* was used for the treatment of cold, headache, rhinitis and toothache as an ethnomedicine in other traditional medicinal systems and countries, such as Korea and Japan (Huang et al., 2022). It is worth noting that *A. dahurica* is also used as a sedative and tonic agent, which is not recorded in traditional medicine book in China (Chung et al., 2012). In Japan, *A. dahurica* is often used to treat skin diseases, such as acne, eruption and erythema. It is also used as an aromatic sedative agent in Japan (Wang et al., 2001).

**TABLE 1:** The traditional uses of *A. dahurica* root in ancient books.

No	Traditional uses	References
1	Treating abnormal leucorrhea in women, pudendal swelling, cold fever and wind evil invading the head and eyes, nourishing the skin	*Shen Nong Ben Cao Jing* (神农本草经) (Dong Han Dynasty, 25–220 A.D.)
2	Curing vomiting, headache with vertigo and itchy eyes	*Ming Yi Bie Lu* (名医别录) (Wei and Jin Dynasty*,* 220–420 A.D.)
3	Treating heart tingling, flooding, hiccup, wind evil, lumbago and apocenosis, brightening eyes and stopping tears	*Yao Xing Lun* (药性论) (Tang Dynasty, 618–907 A.D.)
4	Treating red eyes, pterygium, abortion, mastitis, ulcer in back, scrofula, hematochezia, apocenosis, scabies and lentigo, breaking blood stasis and producing new blood	*Ri Hua Zi Ben Cao* (日华子本草) (Song Dynasty, 960–1279 A.D.)
5	Removing wind of the skin, curing stomach cold, bellyache and cold-damp pain	*Dian Nan Ben Cao* (滇南本草) (Ming Dynasty, 1,368–1644 A.D.)
6	Curing nasosinusitis, epistaxis, toothache, pain in supraorbital bone, constipation, hematuresis, dizzyness, vomiting and sores, antiarsenic poison and snake venom	*Ben Cao Gang Mu* (本草纲目) (Ming Dynasty, 1,368–1644 A.D.)

The clinical application of *A. dahurica* root is also greatly influenced by different processing methods in different ages. In the Southern and Northern Dynasties, stir-baking *A. dahurica* with *Polygonati Rhizoma* can increase the curative effect of *A. dahurica* in treating spleen weakness and dampness obstruction (*Lei Gong Pao Zhi Lun*, Southern and Northern Dynasties, 420–589 A.D.) (雷公炮炙论). In Song Dynasty, there were methods of stewing with wet paper or flour to strengthen the efficacy of *A. dahurica* in treating dampness and diarrhea (*Bo Ji Fang*, Song Dynasty, 960–1279A.D.) (博济方). Stir-baking with blister beetle can enhance the purulent effect of *A. dahurica* (*Chuang Yang Jing Yan Quan Shu*, Song Dynasty, 960–1279 A.D.) (疮疡经验全书). In Yuan Dynasty, the processing methods of stir-frying with vinegar and salt were added to enhance the effects of detumescence and fire elimination of *A. dahurica* (*Shi Yi De Xiao Fang*, Yuan Dynasty, 1,271–1368 A.D.) (世医得效方). Moreover, Char-frying *A. dahurica* can treat female metrorrhagia, and boiling with radish can enhance the properties of *A. dahurica* in dispelling wind and relieving pain (*Ben Cao Gang Mu*, Ming Dynasty, 1,368–1644 A.D.) (本草纲目). Immersing into wine can strengthen the effect of *A. dahurica* in dispelling wind and cold (*Dian Nan Ben Cao*, Ming Dynasty, 1,368–1,644 A.D.) (滇南本草), and immersing into rice water can reduce its dryness (*Ben Cao Meng Quan*, Ming Dynasty, 1,368–1,644 A.D.) (本草蒙筌).

Of note, the root of *A. dahurica* has been used in China for centuries as both a food and traditional medicine. For example, many soups with *A. dahurica* root as ingredient have significant health benefits, such as nourishing blood, warming liver and strengthening kidney. *Bai zhi bo he* liquor (白芷薄荷酒), which is a popular medicinal diet in China, is often used to dispel wind, unblocking stuffy orifice and relieving pain. Interestingly, the root of *A. dahurica* can also be used in cosmetic to improve a person’s skin (Cho et al., 2006). In a word, *A. dahurica* root is a kind of well-known TCM with both food and medicine. Due to its low price and easy availability, many studies indicated that the root of *A. dahurica* should be deeply exploited to treat various diseases and health care.

Additionally, the root of *A. dahurica* is often used in formulas in TCM to cure cold, fever, headache, rheumatic arthritis and other conditions. The well-known prescriptions containing *A. dahurica* root, which have been handed down from many ancient works or ethnic medicine experience are still widely used in modern times (Table 2). Among them, *chuan xiong cha tiao san* (川芎茶调散) is one of the most typical prescriptions to explain the traditional uses of *A. dahurica* root (Chang et al., 2014). The main compositions of the formula include *Ligusticum chuanxiong* Hort, *Nepeta cataria* L, *A. dahurica*, *Notopterygium incisum*, ect. In TCM, *chuan xiong cha tiao san* is used to treat headache, fever and nasal obstruction. In detail, the root of *A. dahurica* is used in *chuan xiong cha tiao san* formula plays an important role in dispelling wind, curing headache and unblocking stuffy nasal cavity. However, few documents provide the chemical composition about the formulas. Consequently, the clinical effects and functions of *A. dahurica* root still need further exploration. The Chinese names of all the medical books and prescriptions are listed in Table 3.

**TABLE 2:** The Prescriptions and traditional uses of *A. dahurica* root in China.

Prescriptions	Main compositions	Traditional Uses	References
*Jiu Wei Qiang Huo Decoction* (九味羌活汤)	Root: *Notopterygium incisum* Ting ex H. T. Chang, *Saposhnikovia divaricate* (Turcz.) Schischk, *Angelica dahurica* **(**Hoffm.) Benth. & Hook.f. ex Franch. & Sav., *Rehmannia glutinosa* Libosch, *Scutellaria baicalensis* Georgi. Rhizome: *Atractylodes Lancea* (Thunb.) DC.*, Ligusticum chuanxiong* Hort. Root and Rhizome: *Asarum sieboldii* Miq, *Glycyrrhiza uralensis* Fisch	Curing cold, rheumatic arthritis, migraine and lumbar muscle strain	*Ci Shi Nan Zhi* (此事难知) (Yuan Dynasty, 1,279–1368 A.D.)
*Chai Ge Jie Ji Decoction* (柴葛解肌汤)	Root: *Bupleurum chinense* DC, *Pueraria lobate* (Willd.) Ohwi, *Scutellaria baicalensis* Georgi, *Notopterygium incisum* Ting ex H. T. Chang, *Angelica dahurica* (Hoffm.) Benth. & Hook.f. ex Franch. & Sav., *Paeonia lactiflora* Pall, *Platycodon grandiflorus* (Jacq.) A. DC. Root and Rhizome: *Glycyrrhiza uralensis* Fisch	Treating fever, headache, nasal cavity dryness, eye pain, pharyngoxerosis, epicophosis, vexation	*Shang Han Liu Shu* (伤寒六书) (Ming Dynasty, 1,368–1644 A.D.)
*Xian Fang Huo Ming Yin* (仙方活命饮)	Root: *Angelica dahurica* (Hoffm.) Benth. & Hook.f. ex Franch. & Sav., *Saposhnikovia divaricate* (Turcz.) *Schischk, Paeonia lactiflora* Pall*, Angelica sinensis* (Oliv.) Diels*, Trichosanthes kirilowii* Maxim*.* Resin: *Boswellia carterii* Birdw*, Commiphora myrrha* Engl*.* Root and Rhizome: *Glycyrrhiza uralensis* Fisch. Flower: *Lonicera Similis* Hemsl. Pericarp: *Citrus reticulata* Blanco*.* Calthrop: *Gleditsia sinensis* L. Scute: *Manis pentadactyia* Linnaeus	Curing carbuncle, turgescence and suppuration	*Jiao Zhu Fu Ren Liang Fang* (校注妇人良方) (Ming Dynasty, 1,368–1644 A.D.)
*Chuan Xiong Cha Tiao San* (川芎茶调散)	Root: *Angelica dahurica* (Hoffm.) Benth. & Hook.f. ex Franch. & Sav., *Notopterygium incisum* Ting ex H. T. Chang, *Saposhnikovia divaricate* (Turcz.) Schischk. Aerial part: *Nepeta cataria* L, *Mentha haplocalyx* Briq. Rhizome: *Ligusticum chuanxiong* Hort. Root and Rhizome: *Glycyrrhiza uralensis* Fisch, *Asarum sieboldii* Miq	Treating headache, fever and nasal obstruction	*Tai Ping Hui Min He Ji Ju Fang* (太平惠民和剂局方) (Song Dynasty, 960–1279 A.D.)
*Da Qin Jiao Decoction* (大秦艽汤)	Root: *Gentiana macrophylla* Pall, *Angelica sinensis* (Oliv.) Diels, *Paeonia lactiflora* Pall, *Notopterygium incisum* Ting ex H. T. Chang, *Saposhnikovia divaricate* (Turcz.) *Schischk, Scutellaria baicalensis* Georgi, *Angelica dahurica* (Hoffm.) Benth. & Hook.f. ex Franch. & Sav., *Rehmannia glutinosa* Libosch, *Heracleum hemsleyanum* Diels. Rhizome: *Ligusticum chuanxiong* Hort, *Atractylodes macrocephala* Koidz. Root and Rhizome: *Glycyrrhiza uralensis* Fisch, *Asarum sieboldii* Miq. Sclerotium: *Poria cocos* (Schw.) Wolf. Gypsum	Curing facial distortion, tongue stiffness and inability to move hands and feet	*Su Wen Bing Ji Qi Yi Bao Ming Ji* (素问病机气宜保命集) (Jin Dynasty, 1,115–1234 A.D.)
*Yu Zhen San* (玉真散)	Root: *Saposhnikovia divaricate* (Turcz.) *Schischk, Angelica dahurica* (Hoffm.) Benth. & Hook.f. ex Franch. & Sav., *Notopterygium incisum* Ting ex H. T. Chang*.* Tuber: *Arisaema erubescens* (Wall.) Schott, *Gastrodia elata* BL, *Typhonium giganteum* Engl	Dispelling wind, relieving pain and stopping spasm	*Wai Ke Zheng Zong* (外科正宗) (Ming Dynasty, 1,368–1644 A.D.)
*Huo Xiang Zheng Qi San* (藿香正气散)	Root: *Angelica dahurica* (Hoffm.) Benth. & Hook.f. ex Franch. & Sav., *Platycodon grandiflorus* (Jacq.) A. DC. Pericarp: *Arecae catechu* L.*, Citrus reticulata* Blanco*.* Fruit: *Perilla frutescens* (L) Britt. Tuber: *Pinellia ternata* (Thunb.) Breit. Rhizome: *Atractylodes macrocephala* Koidz. Root and Rhizome: *Glycyrrhiza uralensis* Fisch. Aerial part: *Pogostemon cablin* (Blanco) Benth. Sclerotium: *Poria cocos* (Schw.) Wolf. Bark: *Magnolia officinalis* Rehd. et Wils	Curing typhoid headache, asthma, cough and spleen–stomach dampness	*Tai Ping Hui Min He Ji Ju Fang* (太平惠民和剂局方) (Song Dynasty, 960–1279 A.D.)
*Jia Wei Wu Ji Yin* (加味五积饮)	Root: *Paeonia lactiflora* Pall, *Angelica dahurica* (Hoffm.) Benth. & Hook.f. ex Franch. & Sav., *Platycodon grandiflorus* (Jacq.) A. DC, *Angelica sinensis* (Oliv.) Diels, *Aucklandia lappa* Decne. Rhizome: *Atractylodes Lancea* (Thunb.) DC.*, Cyperus rotundus* L, *Ligusticum chuanxiong* Hort. Root and Rhizome: *Glycyrrhiza uralensis* Fisch, *Panax ginseng* C. A. Meyer. Pericarp: *Citrus reticulata* Blanco*.* Bark: *Magnolia officinalis* Rehd. et Wils, *Cinnamomum cassia* Presl. Tuber: *Pinellia ternata* (Thunb.) Breit. Fruit:*. Citrus aurantium* L. Herbaceous stem: *Ephedra sinica* Stapf	Treating menstrual cramps	*Nv Ke Zhi Zhang* (女科指掌) (Qing Dynasty, 1,636–1912 A.D.)
*Bai Zhu Shi Hu Decoction* (白术石斛汤)	Root: *Platycodon grandiflorus* (Jacq.) A. DC, *Gentiana macrophylla* Pall, *Angelica dahurica* (Hoffm.) Benth. & Hook.f. ex Franch. & Sav., *Paeonia lactiflora* Pall, *Astragalus membranaceus* (Fisch.) Bunge.*, Angelica sinensis* (Oliv.) Diels. Rhizome: *Atractylodes macrocephala* Koidz. Stem: *Dendrobium nobile* Lindl. Aerial part: *Nepeta cataria* L	Curing qi and blood deficiency, limb burnout and pain of hands and feet	*Sheng Ji Zong Lu* (圣济总录) (Song Dynasty, 960–1279 A.D.)
*Jia Wei Xin Yi San* (加味辛夷散)	Root: *Astragalus membranaceus* (Fisch.) Bunge.*, Angelica sinensis* (Oliv.) Diels, *Paeonia lactiflora* Pall, *Angelica dahurica* (Hoffm.) Benth. & Hook.f. ex Franch. & Sav., *Scutellaria baicalensis* Georgi. Rhizome: *Ligusticum chuanxiong* Hort. Root and Rhizome: *Panax ginseng* C. A. Meyer, *Asarum sieboldii* Miq, *Glycyrrhiza uralensis* Fisch. Bud:*Magnolia denudate* Desr	Treating pus shed from nose	*Xian nian Ji* (仙拈集) (Qing Dynasty, 1,636–1912 A.D.)
*Bai Hu Ge Gen Decoction* (白虎葛根汤)	Root: *Pueraria lobate* (Willd.) Ohwi, *Angelica dahurica* (Hoffm.) Benth. & Hook.f. ex Franch. & Sav., Rhizome: *Anemarrhena asphodeloides* Bunge. Gypsum	Curing headache and fever	*Shang Han Da Bai* (伤寒大白) (Qing Dynasty, 1,636–1912 A.D.)
*Jia Wei Qing Liang Yin* (加味清凉饮)	Root: *Paeonia lactiflora* Pall, *Notopterygium incisum* Ting ex H. T. Chang, *Angelica sinensis* (Oliv.) Diels, *Saposhnikovia divaricate* (Turcz.) Schischk, *Angelica dahurica* (Hoffm.) Benth. & Hook.f. ex Franch. & Sav., *Scutellaria baicalensis* Georgi. Fruit: *Forsythia suspensa* (Thunb.) Vahl, *Gardenia jasminoides* Ellis. Root and Rhizome: *Rheum palmatum* L, *Glycyrrhiza uralensis* Fisch. Aerial part: *Nepeta cataria* L	Treating facial sores	*Song Ya Zun Sheng* (嵩崖尊生) (Qing Dynasty, 1,636–1912 A.D.)

**TABLE 3:** The Chinese names of the medical books and prescriptions.

No	Medical books or prescriptions	Chinese names	Traditional names
1	*Shen Nong Ben Cao Jing*	神农本草经	神農本草経
2	*Ming Yi Bie Lu*	名医别录	名醫别錄
3	*Yao Xing Lun*	药性论	藥性論
4	*Ri Hua Zi Ben Cao*	日华子本草	日華子本草
5	*Dian Nan Ben Cao*	滇南本草	滇南本草
6	*Ben Cao Gang Mu*	本草纲目	本草綱目
7	*Lei Gong Pao Zhi Lun*	雷公炮炙论	雷公炮炙論
8	*Bo Ji Fang*	博济方	博濟方
9	*Chuang Yang Jing Yan Quan Shu*	疮疡经验全书	瘡瘍經驗全書
10	*Shi Yi De Xiao Fang*	世医得效方	世醫得效方
11	*Ben Cao Meng Quan*	本草蒙筌	本草蒙筌
12	*Ci Shi Nan Zhi*	此事难知	此事難知
13	*Shang Han Liu Shu*	伤寒六书	傷寒六書
14	*Jiao Zhu Fu Ren Liang Fang*	校注妇人良方	校注婦人良方
15	*Tai Ping Hui Min He Ji Ju Fang*	太平惠民和剂局方	太平惠民和劑局方
16	*Su Wen Bing Ji Qi Yi Bao Ming Ji*	素问病机气宜保命集	素問病機氣宜保命集
17	*Wai Ke Zheng Zong*	外科正宗	外科正宗
18	*Nv Ke Zhi Zhang*	女科指掌	女科指掌
19	*Sheng Ji Zong Lu*	圣济总录	聖濟總録
20	*Xian nian Ji*	仙拈集	仙拈集
21	*Shang Han Da Bai*	伤寒大白	傷寒大白
22	*Song Ya Zun Sheng*	嵩崖尊生	嵩崖尊生
23	*Jiu Wei Qiang Huo Decoction*	九味羌活汤	九味羌活湯
24	*Chai Ge Jie Ji Decoction*	柴葛解肌汤	柴葛解肌湯
25	*Xian Fang Huo Ming Yin*	仙方活命饮	仙方活命飲
26	*chuan xiong cha tiao san*	川芎茶调散	川芎茶調散
27	*Da Qin Jiao Decoction*	大秦艽汤	大秦艽湯
28	*Yu Zhen San*	玉真散	玉真散
29	*Huo Xiang Zheng Qi San*	藿香正气散	藿香正氣散
30	*Jia Wei Wu Ji Yin*	加味五积饮	加味五積飲
31	*Bai Zhu Shi Hu Decoction*	白术石斛汤	白術石斛湯
32	*Jia Wei Xin Yi San*	加味辛夷散	加味辛夷散
33	*Bai Hu Ge Gen Decoction*	白虎葛根汤	白虎葛根湯
34	*Jia Wei Qing Liang Yin*	加味清凉饮	加味清凉飲
35	*Bai zhi bo he liquor*	白芷薄荷酒	白芷薄荷酒

## 4 Phytochemistry

To date, more than 309 chemical components were isolated and identified from *A. dahurica*. Phytochemical studies have revealed the presence of coumarins, volatile oils, alkaloids, phenols, sterols, benzofurans, polyacetylenes, polysaccharides and others. Currently, studies on the chemical components of *A. dahurica* mostly focus on the root of *A. dahurica*. Coumarins and volatile oils are the predominant constituents of *A. dahurica* root.

### 4.1 Coumarins

Coumarins are the most abundant and main bioactive constituents present in of *A. dahurica.* They have a broad spectrum of pharmacological activities, such as anti-viral (Liu et al., 2021), anti-tumor (Banikazemi et al., 2021), anti-osteoporosis (Jia et al., 2016) and effects on the cardiovascular system (Najmanova et al., 2015). To date, a total of 153 coumarins have been isolated from the root and stem of *A. dahurica*, include 18 simple coumarins (**1–18**), 93 furanocoumarins (**19–111**), 41 coumarins glycosides (**112–147**), 3 other coumarins (**148–150**) and 3 coumarin derivatives (**151–153**). Among them, furanocoumarins are the most abundant coumarins, which are mainly divided into linear and angular types based on the location of the furan group. The furan ring in linear furocoumarins is connected to the 6 and 7 carbon atoms, while the substituent often occurs in the positions of C_7_ and C_8_ in angelic furocoumarins (Sumorek-Wiadro et al., 2020). Furanocoumarin IMP (22) is the most principal and representative active component of *A. dahurica* root, which has anti-inflammatory, analgesic, anti-allergic and neuroprotective activities (Deng et al., 2020). Moreover, Other furanocoumarins such as isoimperatorin (**19**), oxypeucedanin (**20**), phellopterin (**26**) and byakangelicin (**41**) are also characteristic constituents of *A. dahurica* root with a wide range of bioactivities (Cho et al., 2006; Kang et al., 2008; Lee, B.W. et al., 2020; Li and Wu, 2017). These furanocoumarins are charicterized by the attachment of different substituents to C_5_ or C_8_ in the parent nucleus of linear furocoumarins. In addition to furanocoumarins, some simple coumarins, such scopletin (**3**) in the root and stem of *A. dahurica* also exhibited anti-microbial and neuroprotective effects (Kwon et al., 1997; Luo et al., 2020), which contribute to the bioactivities of *A. dahurica*. The information and chemical structures of all these coumarins are listed in Supplementary Table S1 and Supplementary Figure S1.

### 4.2 Volatile Oils

Volatile oils are other major physiologically active compounds in *A. dahurica*. The components of volatile oil can be roughly divided into four categories, including terpenoids, aromatic compounds, aliphatic compounds and other compounds. Among them, terpenoids are the most common type. Numerous studies declared that the cluster of the compounds possess extensive bioactivities and act as antibacterials, antivirals and insecticides in plants (Cascaes et al., 2021). So far, approximately 121 volatile components have been identified from the root of *A. dahurica*. These volatile oils include terpenes (**154–200**), aromatics (**201–212**), alcohols (**213–234**), aldehydes (**235–246**), ketones (**247–252**), acids (**253–260**), esters (**261–270**) and alkanes (**271–274**). The major components of the volatile oils in *A. dahurica* root include α-pinene (**154**), myrcene (**199**), terpinen-4-ol (**219**), 1-dodecanol (**221**) and sabinene (**160**). However, the extraction yields of volatile oil are different as the plant materials came from different regions. It was reported that the extraction yield of volatile oil from *A. dahurica* root cultivated in yuzhou, China is 1.4% (ml/g), including α-pinene (44.91%), myrcene (8.72%), terpinen-4-ol (8.01%), 1-dodecanol (6.43%) and sabinene (3.42%). These volatile oils were confirmed to show obvious antioxidant activity (Wang D. et al., 2020). All the identified volatile oils are listed in Table 4 and their structural formulas are displayed in Supplementary Figure S2.

**TABLE 4:** Volatile oils isolated from *A. dahurica*.

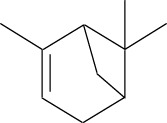	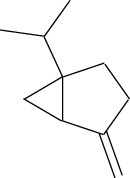	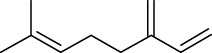	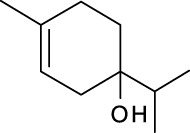	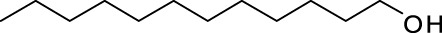
α-pinene (154)	Sabinene (160)	Myrcene (199)	Terpinen-4-ol (219)	1-Dodecanol (221)
No	Names	Plant pats	Formulas	References
Terpenes				
154	α-Pinene	Roots	C_10_H_16_	Sun et al. (2017)
155	1-Caryophyllene	Roots	C_15_H_24_	Sun et al. (2017)
156	E-1,9-tetradecadiene	Roots	C_14_H_26_	Sun et al. (2017)
157	1-Methylcyclooctene	Roots	C_9_H_16_	Sun et al. (2017)
158	Camphene	Roots	C_10_H_16_	Hu et al. (2019)
159	β-Pinene	Roots	C_10_H_16_	Hu et al. (2019)
160	Sabinene	Roots	C_10_H_16_	Hu et al. (2019)
161	β-Myrcene	Roots	C_10_H_16_	Hu et al. (2019)
162	α-Phellandrene	Roots	C_10_H_16_	Hu et al. (2019)
163	α-Terpinene	Roots	C_10_H_16_	Hu et al. (2019)
164	D-Limonene	Roots	C_10_H_16_	Hu et al. (2019)
165	β-Phellandrene	Roots	C_10_H_16_	Hu et al. (2019)
166	Eucalyptol	Roots	C_10_H_18_O	Hu et al. (2019)
167	γ-Terpinene	Roots	C_10_H_16_	Hu et al. (2019)
168	Trans-β-Ocimene	Roots	C_10_H_16_	Hu et al. (2019)
169	α-Copaene	Roots	C_15_H_24_	Hu et al. (2019)
170	β-Cubebene	Roots	C_15_H_24_	Hu et al. (2019)
171	Selina-5,11-diene	Roots	C_15_H_24_	Hu et al. (2019)
172	Longifolene-(V4)	Roots	C_15_H_24_	Hu et al. (2019)
173	(-)-β-Elemene	Roots	C_15_H_24_	Hu et al. (2019)
174	Caryophyllene	Roots	C_15_H_24_	Hu et al. (2019)
175	Aromandendrene	Roots	C_15_H_24_	Hu et al. (2019)
176	γ-Elemene	Roots	C_15_H_24_	Hu et al. (2019)
177	cis-β-Farnesene	Roots	C_15_H_24_	Hu et al. (2019)
178	Humulene	Roots	C_15_H_24_	Hu et al. (2019)
179	γ-Muurolene	Roots	C_15_H_24_	Hu et al. (2019)
180	δ-Elemene	Roots	C_15_H_24_	Hu et al. (2019)
181	β-selinene	Roots	C_15_H_24_	Hu et al. (2019)
182	δ-Cadinene	Roots	C_15_H_24_	Hu et al. (2019)
183	α-Gurjunene	Roots	C_15_H_24_	Hu et al. (2019)
184	α-Guaiene	Roots	C_15_H_24_	Hu et al. (2019)
185	γ-Selinene	Roots	C_15_H_24_	Hu et al. (2019)
186	β-Guaiene	Roots	C_15_H_24_	Hu et al. (2019)
187	Germacrene B	Roots	C_15_H_24_	Hu et al. (2019)
188	Caryophyllene oxide	Roots	C_15_H_24_O	Hu et al. (2019)
189	α-thujene	Roots	C_10_H_16_	Tabanca et al. (2014)
190	Limonene	Roots	C_10_H_16_	Tabanca et al. (2014)
191	Perillen	Roots	C_10_H_14_O	Tabanca et al. (2014)
192	Trans-β-bergamotene	Roots	C_15_H_24_	Tabanca et al. (2014)
193	Selina-4,11-diene	Roots	C_15_H_24_	Tabanca et al. (2014)
194	β-Bisabolene	Roots	C_15_H_24_	Tabanca et al. (2014)
195	α-Selinene	Roots	C_15_H_24_	Tabanca et al. (2014)
196	Selina-4(15),7(11)-diene	Roots	C_15_H_24_	Tabanca et al. (2014)
197	Humulene epoxide II	Roots	C_15_H_24_O	Tabanca et al. (2014)
198	δ-3-Carene	Roots	C_10_H_16_	Dongying Wang et al. (2020)
199	Myrcene	Roots	C_10_H_16_	Dongying Wang et al. (2020)
200	Sesquisabinene	Roots	C_15_H_24_	Dongying Wang et al. (2020)
Aromatics				
201	1-Methoxy-4-[(Z)-prop-1-enyl]benzene	Roots	C_10_H_12_O	Sun et al. (2017)
202	*p*-Cymene	Roots	C_10_H_14_	Hu et al. (2019)
203	Toluene	Roots	C_7_H_8_	Hu et al. (2019)
204	1,3-dimethylbenzene	Roots	C_8_H_10_	Hu et al. (2019)
205	*o*-Xylene	Roots	C_8_H_10_	Hu et al. (2019)
206	α-p-Dimethylstyrene	Roots	C_10_H_12_	Hu et al. (2019)
207	α,3-Dimethylstyrene	Roots	C_10_H_12_	Hu et al. (2019)
208	Estragole	Roots	C_10_H_12_O	Hu et al. (2019)
209	Anethole	Roots	C_10_H_12_O	Hu et al. (2019)
210	Isoelemicin	Roots	C_12_H_16_O_3_	Hu et al. (2019)
211	Cuparene	Roots	C_15_H_22_	Tabanca et al. (2014)
212	Carvacrol methyl ether	Roots	C_11_H_16_O	Dongying Wang et al. (2020)
Alcohols				
213	Dodecyl alcohol	Roots	C_12_H_26_O	Sun et al. (2017)
214	1-Pentadecanol	Roots	C_15_H_32_O	Sun et al. (2017)
215	Linalool	Roots	C_10_H_18_O	Hu et al. (2019)
216	3-Buten-2-ol, 2-methyl-	Roots	C_5_H_10_O	Hu et al. (2019)
217	Prenol	Roots	C_5_H_10_O	Hu et al. (2019)
218	1-Hexanol	Roots	C_6_H_14_O	Hu et al. (2019)
219	Terpinen-4-ol	Roots	C_10_H_18_O	Hu et al. (2019)
220	Benzyl alcohol	Roots	C_7_H_8_O	Hu et al. (2019)
221	1-Dodecanol	Roots	C_12_H_26_O	Hu et al. (2019)
222	1-Hexadecanol	Roots	C_16_H_34_O	Hu et al. (2019)
223	Spathulenol	Roots	C_15_H_24_O	Hu et al. (2019)
224	2-Methyl-3-buten-2-ol	Roots	C_5_H_10_O_5_	Tabanca et al. (2014)
225	cis-p-Menth-2-en-1-ol	Roots	C_10_H_18_O	Tabanca et al. (2014)
226	trans-Pinocarveol	Roots	C_10_H_16_O	Tabanca et al. (2014)
227	cis-Piperitol	Roots	C_10_H_18_O	Tabanca et al. (2014)
228	Myrtenol	Roots	C_10_H_16_O	Tabanca et al. (2014)
229	p-Mentha-1,5-dien-7-ol	Roots	C_10_H_16_O	Tabanca et al. (2014)
230	p-Cymen-8-ol	Roots	C_10_H_14_O	Tabanca et al. (2014)
231	1-Tridecanol	Roots	C_13_H_28_O	Tabanca et al. (2014)
232	Cumin alcohol	Roots	C_10_H_14_O	Tabanca et al. (2014)
233	Guaia-6,10 (14)-dien-4β-ol	Roots	C_15_H_24_O	Tabanca et al. (2014)
234	2-Nonanol	Roots	C_9_H_20_O	Dongying Wang et al. (2020)
Aldehydes				
235	Butanal, 3-methyl-	Roots	C_5_H_10_O	Hu et al. (2019)
236	Hexanal	Roots	C_6_H_12_O	Hu et al. (2019)
237	2-Methyl-2-butenal	Roots	C_5_H_8_O	Hu et al. (2019)
238	Heptanal	Roots	C_7_H_14_O	Hu et al. (2019)
239	Octanal	Roots	C_8_H_16_O	Hu et al. (2019)
240	(E)-2-Octenal	Roots	C_8_H_14_O	Hu et al. (2019)
241	Nonanal	Roots	C_9_H_18_O	Hu et al. (2019)
242	Decanal	Roots	C_10_H_20_O	Hu et al. (2019)
243	Benzaldehyde	Roots	C_7_H_6_O	Hu et al. (2019)
244	(E)-2-Nonenal	Roots	C_9_H_16_O	Hu et al. (2019)
245	2,6-Octadienal,3,7-dimethyl-, (Z)-	Roots	C_10_H_16_O	Hu et al. (2019)
246	Cumin aldehyde	Roots	C_10_H_12_O	Tabanca et al. (2014)
Ketones				
247	6-Methyl-5-hepten-2-one	Roots	C_8_H_14_O	Hu et al. (2019)
248	2-Nonanone	Roots	C_9_H_18_O	Hu et al. (2019)
249	Camphor	Roots	C_10_H_16_O	Hu et al. (2019)
250	Pinocarvone	Roots	C_10_H_14_O	Tabanca et al. (2014)
251	Cryptone	Roots	C_9_H_14_O	Tabanca et al. (2014)
252	Verbenone	Roots	C_10_H_14_O	Tabanca et al. (2014)
Acids				
253	Tridecanoic acid	Roots	C_13_H_26_O_2_	Sun et al. (2017)
254	Linoleic acid	Roots	C_18_H_32_O_2_	Sun et al. (2017)
255	Palmitic acid	Roots	C_16_H_32_O_2_	Zhang et al. (2018)
256	Stearic acid	Roots	C_18_H_36_O_2_	Zhang et al. (2018)
257	Acetic acid	Roots	C_2_H_4_O_2_	Hu et al. (2019)
258	Hexanoic acid	Roots	C_6_H_12_O_2_	Hu et al. (2019)
259	Oleic Acid	Roots	C_18_H_34_O_2_	Hu et al. (2019)
260	Dodecanoic acid	Roots	C_12_H_24_O_2_	Hu et al. (2019)
Esters				
261	Oxacyclotetradecan-2-one	Roots	C_13_H_24_O_2_	Sun et al. (2017)
262	Hexadecanoic acid, ethyl ester	Roots	C_18_H_36_O_2_	Sun et al. (2017)
263	2-Ethylhexyl hydrogen phthalate	Roots	C_16_H_22_O_4_	Sun et al. (2017)
264	Ethyl 15-methylheptadecanoate	Roots	C_20_H_40_O_2_	Sun et al. (2017)
265	Linoleic acid ethyl ester	Roots	C_20_H_36_O_2_	Sun et al. (2017)
266	Cyclopropanecarboxylic acid,3-methylphenyl ester	Roots	C_11_H_12_O_2_	Sun et al. (2017)
267	Ethyl oleate	Roots	C_20_H_38_O_2_	Sun et al. (2017)
268	Vinyl acetate	Roots	C_4_H_6_O_2_	Hu et al. (2019)
269	ethyl-(E)-cinnamate	Roots	C_11_H_12_O_2_	Hu et al. (2019)
270	γ-Decalactone	Roots	C_10_H_18_O_2_	Hu et al. (2019)
Alkanes				
271	Cyclododecane	Roots	C_12_H_24_	Sun et al. (2017)
272	Heptatriacontane	Roots	C_37_H_76_	Qiao et al. (1996)
273	Undecane	Roots	C_11_H_24_	Dongying Wang et al. (2020)
274	Tridecane	Roots	C_13_H_28_	Dongying Wang et al. (2020)

### 4.3 Alkaloids

Biologically important alkaloids have been less distributed in *A. dahurica*. Approximately 13 types of alkaloids have been isolated from this plant (Table 5 and Supplementary Figure S3), including dahurines A–F (**275–280**), (8R,11S,12R)-Funebral (**281**), (8R,11S,12R)-3,4-dihydro-3-amino-4,5-dimethylfuran-2 [5H]-one-2-formyl pyrrole (**282**), 4″-butyl-2-formyl-5-(hydroxymethyl)-1H-pyrrole-1-butanoic acid (**283**), butyl 2-formyl-5-butoxymethyl-1H-pyrrole-1-butanoate (**284**), hemerocallisamine II (**285**), butyl 2-pyrrolidone-5-carboxylate (**286**) and corydaldine (**287**) (Sun et al., 2017; Qi et al., 2019).

**TABLE 5:** Alkaloids, phenols, sterols, benzofurans and polyacetylenes isolated from *A. dahurica*.

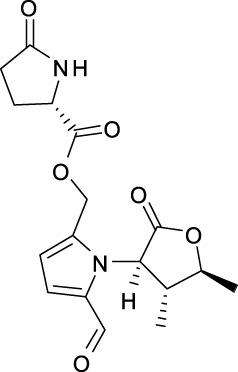	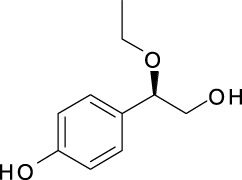	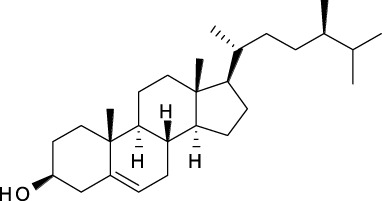	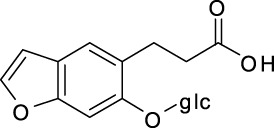	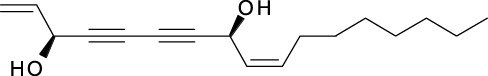
Dahurine A (275)	Angelicols A (288)	β-Sitosterol (299)	Cnidioside A (304)	Falcarindiol (307)
No	Names	Plant parts	Formulas	References
Alkaloids				
275	Dahurine A	Roots	C_17_H_20_N_2_O_6_	Qi et al. (2019)
276	Dahurine B	Roots	C_16_H_23_NO_4_	Qi et al. (2019)
277	Dahurine C	Roots	C_16_H_23_NO_4_	Qi et al. (2019)
278	Dahurine D	Roots	C_12_H_15_NO_3_	Qi et al. (2019)
279	Dahurine E	Roots	C_23_H_31_NO_4_	Qi et al. (2019)
280	Dahurine F	Roots	C_17_H_27_NO_4_	Qi et al. (2019)
281	(8*R*,11*S*,12*R*)-Funebral	Roots	C_12_H_17_NO_4_	Qi et al. (2019)
282	(8*R*,11*S*,12*R*)-3,4-dihydro-3-amino-4,5-dimethylfuran-2 [5*H*]-one-2-formyl pyrrole	Roots	C_11_H_14_NO_3_	Qi et al. (2019)
283	4″-Butyl-2-formyl-5-(hydroxymethyl)-1*H*-pyrrole-1-butanoic acid	Roots	C_14_H_21_NO_4_	Qi et al. (2019)
284	Butyl 2-formyl-5-butoxymethyl-1*H*-pyrrole-1-butanoate	Roots	C_18_H_31_NO_4_	Qi et al. (2019)
285	Hemerocallisamine II	Roots	C_10_H_17_NO_2_	Qi et al. (2019)
286	Butyl 2-pyrrolidone-5-carboxylate	Roots	C_9_H_15_NO_3_	Qi et al. (2019)
287	Corydaldine	Roots	C_11_H_13_NO_3_	Sun et al. (2017)
Phenols				
288	Angelicols A	Roots	C_10_H_14_O_3_	Shu et al. (2020a)
289	Angelicols B	Roots	C_18_H_20_O_6_	Shu et al. (2020a)
290	(1S)-2-O-Z-Feruloyl-1-(4-hydroxyphenyl)ethane-1,2-diol	Roots	C_18_H_18_O_6_	Shu et al. (2020a)
291	(1S)-2-O-E-Feruloyl-1-(4-hydroxyphenyl)ethane-1,2-diol	Roots	C_18_H_18_O_6_	Shu et al. (2020a)
292	Ferulic acid	Roots	C_10_H_10_O_4_	Kwon et al. (1997)
293	Cyanidin	Roots	C_15_H_11_O_6_	Pervin et al. (2014)
294	Rutin	Roots	C_27_H_30_O_16_	Pervin et al. (2014)
295	Catechin	Roots	C_15_H_14_O_6_	Pervin et al. (2014)
296	Epicatechin	Roots	C_15_H_14_O_6_	Pervin et al. (2014)
297	Kaempferol	Roots	C_15_H_10_O_6_	Pervin et al. (2014)
298	4-O-β-D-Glucopyranosyl-9-O-β-D-glucopyranosyl-(7R,8S)-dehydrodiconiferyl alcohol	Roots	C_32_H_42_O_16_	Zhao et al. (2007a)
Sterols				
299	β-Sitosterol	Roots	C_29_H_50_O	Li and Wu, (2017)
300	Daucosterol	Roots	C_35_H_60_O_6_	Li and Wu, (2017)
Benzofurans				
301	3-[6,7-Furano-9-hydroxy-4-(2″,3″-dihydroxy-3″-methylbutyloxy)]-phenyl propionic acid	Roots	C_16_H_20_O_7_	Matsuo et al. (2020)
302	3-[6,7-Furano-9-(β-D-glucopyranosyloxy)-4-(2″,3″-dihydroxy-3″-methylbutyloxy)]-phenyl propionic acid	Roots	C_22_H_30_O_12_	Matsuo et al. (2020)
303	3-[6,7-Furano-9-(β-Dglucopyranosyloxy)-4-(2″,3″-dihydroxy-3″-methylbutyloxy)]-phenyl propionic acid methyl ester	Roots	C_23_H_32_O_12_	Matsuo et al. (2020)
304	Cnidioside A	Roots	C_17_H_20_O_9_	Matsuo et al. (2020)
305	Methylcnidioside A	Roots	C_18_H_22_O_9_	Matsuo et al. (2020)
306	Methylpicraquassioside	Roots	C_19_H_24_O_10_	Matsuo et al. (2020)
Polyacetylene				
307	Falcarindiol	Roots	C_17_H_24_O_2_	Choi et al. (2005)
308	Octadeca-1,9-dien-4,6-diyn-3,8,18-triol	Roots	C_18_H_26_O_3_	Choi et al. (2005)
Other compounds				
309	Adenosine	Roots	C_10_H_13_N_5_O_4_	Shu et al. (2020b)

### 4.4 Phenols

There have been four phenolic compounds identified from the ethanol extract of *A. dahurica* root, including angelicols A (**288**), angelicols B (**289**), (1S)-2-O-Z-Feruloyl-1-(4-hydroxyphenyl)ethane-1,2-diol (**290**) and (1S)-2-O-E-Feruloyl-1-(4-hydroxyphenyl)ethane-1,2-diol (**291**) (Shu et al., 2020a). Another phenolic compound, ferulic acid (**292**) was isolated from the EtOAc-soluble fraction of *A. dahurica* root (Kwon et al., 1997). In addition, five flavonoids, including cyanidin **(293)**, rutin **(294)**, catechin **(295)**, epicatechin **(296)** and kaempferol **(297)** have been found in the water extract or ethanol extract (Pervin et al., 2014). Zhao X. Z. et al. (2007) reported a kind of new neolignan glycoside, namely 4-O-β-D-glucopyranosyl-9-O-β-D-glucopyranosyl-(7R,8S)-dehydrodiconiferyl alcohol (**298**) from the fresh root of *A. dahurica*. The information of them is shown in Table 5 and Supplementary Figure S3.

### 4.5 Sterols

Sterols such as β-sitosterol (**299**) and daucosterol (**300**) were identified from the root of *A. dahurica* (Table 5 and Supplementary Figure S3) (Li and Wu, 2017). Plant sterols have been reported to reduce the circulating total cholesterol (TC) and low density lipoprotein cholesterol (LDL-C) to prevent cardiovascular disease (Chen et al., 2019). Although these compounds have been shown to be potential and safe drugs by many *in vitro* and *in vivo* studies, clinical studies are needed to prove the implications of these compounds on some specific diseases so as to develop them into notable drugs (Babu and Jayaraman, 2020).

### 4.6 Benzofurans

Benzofurans are an important class of heterocyclic compounds, which are diffusely presented in natural products and synthetic materials (Khodarahmi et al., 2015). In a recent study, six benzofuran derivatives have been acquired from the root of *A. dahurica* (Table 5 and Supplementary Figure S3), including 3-[6,7-furano-9-hydroxy-4-(2″,3″-dihydroxy-3″-methylbutyloxy)]-phenyl propionic acid (**301**), 3-[6,7-furano-9-(β-D-glucopyranosyloxy)-4-(2″,3″-dihydroxy-3″-methylbutyloxy)]-phenyl propionic acid (**302**), 3-[6,7-furano-9-(β-Dglucopyranosyloxy)-4-(2″,3″-dihydroxy-3″- methylbutyloxy)]-phenyl propionic acid methyl ester (**303**), cnidioside A (**304**), methylcnidioside A (**305**) and methylpicraquassioside (**306**) (Matsuo et al., 2020).

### 4.7 Polyacetylenes

Polyacetylenes are pervasively found in the family Asteraceae, Araliaceae and Apiaceae (Lin et al., 2016). Up to now, only two polyacetylenes, falcarindiol (**307**) and octadeca-1,9-dien-4,6-diyn-3,8,18-triol (**308**) have been reported from the root of *A. dahurica* (Table 5 and Supplementary Figure S3) (Choi et al., 2005).

### 4.8 Polysaccharides

*A. dahurica* polysaccharides have also been reported in some research publications. A latest study reported a new acidic *A. dahurica* polysaccharide (ADP) composed of rhamnose, mannose, glucose, galactose, arabinose, galacturonic acid and glucuronic acid with a Mw of 6.09 × 10^3^ Da (Dong et al., 2021). Xu et al. (2011) isolated four ADPs from the water extract of *A. dahurica* root and found that they have different degrees of anti-oxidant activity. Moreover, Wang et al. (2021) isolated a gluco-arabinan consisting of a trace of glucose and arabinose with a Mw of 9,950 Da by water extraction and ethanol precipitation from the root of *A. dahurica*.

### 4.9 Other Compounds

In addition to the compounds mentioned above, adenosine (**309**) was also isolated from the root of *A. dahurica* (Table 5 and Supplementary Figure S3) (Shu et al., 2020b). Moreover, *A. dahurica* also contains sucrose and amino acids (Zhao and Yang, 2018).

## 5 Pharmacology

As of the present, a strong body of evidence for the bioacitivities of *A. dahurica* has been discovered. The crude extract and active components of *A. dahurica* contain various bioactivities, such as anti-inflammation, anti-tumor, anti-oxidation, analgesic activity, antiviral and anti-microbial effects, effects on the cardiovascular system, neuroprotective function, hepatoprotective activity, effects on skin diseases and so on. These biological activities have proved most implications of *A. dahurica* root in treating cold, headache, toothache, cold-damp pain, rhinitis and skin diseases. Next, these bioactivities were discussed and the recapitulative summary was listed in Supplementary Table S2.

### 5.1 Anti-Inflammatory Activity

#### 5.1.1 Crude Extracts

Nowadays, there have been growing evidence showing that *A. dahurica* has been widely used for inflammation-associated diseases. For example, the 50% ethanol extract of *A. dahurica* root showed a significant inhibitory effect on lipopolysaccharide (LPS)-induced inflammation in Raw 264.7 cells (10 and 100 μg/ml for 2 h) and rat models of periodontitis (1 and 100 mg/ml for 14 days). The expression of inflammatory genes, including interleukin-1β (IL-1β), IL-6, IL-8 and interferon-γ (IFN-γ) were decreased in gingival tissues of ligature-induced periodontitis rats and LPS-induced Raw 264.7 cells upon treatments with ethanol extract of *A. dahurica*. Moreover, the extract of *A. dahurica* root inhibited the expression of nuclear factor-κB (NF-κ B), cyclooxygenase-2 (COX-2) and inducible nitric oxide synthase (iNOS), and the phosphorylation of inhibitor of NF-κB (IκB). Therefore, the anti-inflammatory effects of *A. dahurica* in periodontitis might occur via the regulation of pro-inflammatory mediators (Lee et al., 2017). In asthmatic mice, the 70% ethanol extract of *A. dahurica* root (50 and 100 mg/kg b. w., 5 days) relieved ovalbumin-induced airway inflammation, as evidenced by the reduction of eosinophilia, cytokines (IL-4, IL-5), tumor necrosis factor-α (TNF-α), immunoglobulin E (Ig E) and mucus production by increasing the expression of heme oxygenase-1 (HO-1) (Lee et al., 2011).

#### 5.1.2 Isolated Compounds

What’s more, many compounds isolated from *A. dahurica* also possess excellent anti-inflammatory properties. For example, the administration of IMP (15, 30 and 60 mg/kg b. w., 7 days), which is the most major ingredient of *A. dahurica*, significantly inhibited the ear edema of dimethylbenzene-induced mice, acetic acid-induced vascular permeability in mice and ball fralunoma weight cotton pellet-induced granuloma in rats. Further investigation demonstrated that IMP reduced the levels of TNF-α, IL-6, IL-1β, iNOS and COX-2 in LPS-induced RAW 264.7 cells by suppressing the activity of NF-κ B *via* increasing the expression of p65 (C) and IκB (C) and decreasing the level of p65 (N) (Zhang X. et al., 2017). Li et al. isolated 13 coumarins from the root of *A. dahurica* and evaluated their abilities of anti-allergic inflammation. They found that all these coumarins at a dose of 20 μM for 1 h could reduce the release of histamine in the media for RBL-2H3 cells compared with dinitrophenyl-human serum albumin (DNP-HSA) cells, with oxypeucedanin hydrate (**21**), bergapten (**25**) and byakangelicin (**41**) possessing the strongest property. Moreover, these compounds reduced the secretion of TNF-α, IL-4 and IL-1β, with bergapten and phellopterin (**26**) exhibiting the most potent effect. The treatment mechanism might be the inhibition of NF-κ B signaling (Li and Wu, 2017).

In summary, the related results showed that both crude extracts and active compounds of *A. dahurica* exhibit significant anti-inflammatory activity, and their mechanism is mainly through inhibiting the exression and release of pro-inflammatory mediators, such as NF-κ B, iNOS, COX-2 and TNF-α etc. This activity may link to the traditional uses of *A. dahurica* root in treating cold, toothache, rhinitis and some skin diseases.

### 5.2 Anti-Tumor Activity

Modern pharmacological studies have revealed that *A. dahurica* also exhibits potent anti-tumor effects in multiple cancers, including colon cancer, breast cancer and melanoma. In murine melanoma B16F10 cells, the 70% ethanol extract of *A. dahurica* root (100 and 200 μg/ml for 24 h) was confirmed to inhibit the growth, migration, invasion and colony formation, while stimulating cell apoptosis via reducing the activity of matrix metalloproteinase-2 (MMP-2) and MMP-9 (Hwangbo et al., 2020). The essential oils from the root of *A. dahurica* (12.5 μg/ml for 24 h) could suppress the resistance of MCF-7/ADR breast cancer cells to doxorubicin with a fold reversal of 2.09 by inhibiting the expression of ATP-binding cassette subfamily B member1 (ABCB1) and decreasing lipid raft stability (Wu et al., 2016). As for colon cancer, the cell apoptosis assay illustrated anti-apoptosis effect of the ethyl acetate extract of *A. dahurica* root (200 and 250 μg/ml, 48 h) on colon cancer HT-29 cells through p53-independent pathway (Zheng et al., 2016b). Moreover, IMP was reported to significantly inhibited the proliferation at a dose of 150 μM for 12 h in colon cancer HCT116 cells, as well as suppressed angiogenesis and tumor growth (50 and 100 mg/kg b. w., 3 times a week, 35 days) in HCT116 xenograft mice by inhibiting hypoxia-inducible factor-1α (HIF-1α) protein synthesis through the mammalian target of rapamycin (mTOR)/ribosomal protein S6 kinase (p70S6K)/eukaryotic initiation factor 4E binding protein-1 (4E-BP1) and mitogen-activated protein kinase (MAPK) signaling pathways (Mi et al., 2017). It could also significantly suppress the growth (IC_50_ = 78 μM), and induce the apoptosis at a dose of 150 μM for 48 h in colon cancer HT-29 cells via upregulating p53 and caspase cascade (Zheng et al., 2016a). In addition, IMP enhanced anokis at doses of 0.1, 0.5 and 1 μg/ml in lung cancer H292 and A549 cells at 24 h after cell detachent and mitigated cancer cachexia at doses of 25 and 50 mg/kg b. w. for 15 days in colorectal adenocarcinoma CT26 tumor-bearing mice (Choochuay et al., 2013; Chen et al., 2020). These results suggested that IMP, the active ingredient of *A. dahurica*, is a new potential candidate for cancer treatment.

Although emerging evidence has demonstrated the anti-tumor effect of *A. dahurica*, several challenges must be overcome in the future. Firstly, the pathogenesis of tumors is complex and the research on the anti-tumor mechanism of *A. dahurica* is not in-depth enough. Furthermore, many studies focused on the crude extracts and could not determine the specific ingredient in *A. dahurica* that was responsible for its anti-tumor activity. Finally, the current studies mainly include *in vivo* and *in vitro* experiments, with a lack of clinical trial data. Future studies are necessary to reveal the anti-tumor effect of *A. dahurica* in clinical trial.

### 5.3 Anti-Oxidant Activity

#### 5.3.1 Crude Extracts

The *A. dahurica* extract exerted significant anti-oxidant activity mainly based on its free radicals scavenging ability (Lee and Woo, 2011; Wang et al., 2017; Liang et al., 2018). Wang et al. (2017) assessed the anti-oxidant activities of different extracts of the root of *A. dahurica* and found that 70% ethanol extract displayed the most powerful anti-oxidant with 50% inhibitory concentration (IC_50_) of 1.6 ± 0.25 mg/ml using 1,1-diphenyl-2-picrylhydrazyl (DPPH) assay. Similarly, 70% ethanol extract of *A. dahurica* root exhibited the highest reducing power (IC_50_ = 2.8 ± 0.36 mg/ml) compared with water extract and ethyl acetate extract. Interestingly, they also found that their anti-oxidant activities were improved after fermentation by probiotic bacteria. Lee et al. found that both extracts of *A. dahurica* stem (including leaves) and root exhibited anti-oxidant effect. The DPPH radical scavenging acivity of stem [50% effective concentration (EC_50_) = 243.33 μg/ml] was more powerful than that of root (EC_50_ = 1,161.79 μg/ml), while the xanthine oxidase inhibitory activities of them showed no significant differences with EC_50_ values of 434.66 μg/ml and 435.19 μg/ml, respectively (Lee and Woo, 2011). These results indicated that both the stem and root extracts of *A. dahurica* have certain anti-oxidant effect. Nevertheless, the DPPH analytical method may overestimate the anti-oxidant content and this method cannot test all the analytical properties of the extract. Thus, the anti-oxidant activity cannot be accurately evaluated only by DPPH analysis and it is necessary to try a more precise method to verify it.

#### 5.3.2 Isolated Compounds

In addition to the crude extract of *A. dahurica*, some chemical components from *A. dahurica*, including coumarins, phenols and polysaccharides also possess obvious anti-oxidant activities (Piao et al., 2004; Xu et al., 2011; Bai et al., 2016; Kang et al., 2019; Shu et al., 2020a). For example, Piao et al. identified 11 furanocoumarins from the root of *A. dahurica* and found that 9-hydroxy-4-methoxypsoralen (**67**) and alloisoimperatorin (**107**) significantly attenuated 2,2-azobis (2-aminodinopropane)-dihydrochloride (AAPH)-induced renal epithelial cell injury by reducing DPPH radical with IC_50_ of 6.1 and 9.4 μg/ml (Piao et al., 2004). Phenols compounds (**289, 290** and **291**) from *A. dahurica* root show significant DPPH radical scavenging activities with IC_50_ of 0.36, 0.39 and 0.44 mM (Shu et al., 2020a). Moreover, the polysaccharides ADP1-ADP4 from the root of *A. dahurica* also exhibit powerful anti-oxidant capacity at doses ranging from 62.5 to 500 μg/ml by inhibiting malondialdehyde (MDA) formation and chelating ferrous ion (Fe^2+^) (Xu et al., 2011). These findings showed that coumarins, phenols and polysaccharides in *A. dahurica* exhibit antioxidant effect. However, *in vitro* experiments used to test anti-oxidant activity are prone to interference and further *in vivo* experiments are required to confirm these results.

### 5.4 Analgesic Activity

*A. dahurica* has been used historically to cure pain-associated diseases, such as headache, toothache, rheumatalgia and superciliary ridge pain. Modern molecular pharmacological approaches have demonstrated the analgesic effects of *A. dahurica* root by using multiple pain models and revealed that the analgesic mechanisms are complex. Transient receptor potential vanilloid type 1 (TRPV1) is a therapeutic target for treating various models of pain and is widely expressed in peripheral and central nervous systems (Iftinca et al., 2021). Recently, the researchers indicated that injected subcutaneously with IMP (2.45 mM) could effectively alleviated the acute pain induced by formalin or capsaicin in rats by inhibiting the activity of TRPV1 channel (Chen et al., 2014). Similarly, The water extract of *A. dahurica* root at a dose of 100 mg/kg b. w. for 2 h attenuated the acute pain induced by thermal, formalin and capsaicin in mice through the inhibition of TRPV1 channel (Guo et al., 2019). Moreover, coumarins of *A. dahurica* root (CAD) obviously reduced the nociceptive response at doses 30, 60 and 120 mg/kg b. w. for 4 days in formalin-induced pain models of mice. After intracerebroventricular administration of CAD at dose 6 mg/kg b. w., the latency of mice was significantly prolonged in the hotplate test. Further research suggested that the analgesic site of CAD might be in both peripheral and central nervous systems, and the mechanism might be associated with the synthesis and release of nitric oxide (NO) (Wang H.L. et al., 2009). These findings demonstrated that the extracts of *A. dahurica* root or individual compound may exert analgesic effect by the inhibiting TRPV1 channel and regulating NO level. The root of *A. dahurica* might have the potential to be effective therapeutic drug for various pains, which was consistent with its traditional use of analgesia.

### 5.5 Antiviral and Anti-Microbial Activities

Nowadays, some studies revealed that *A. dahurica* present a wide range of anti-microbial activity. Kwon et al. first isolated eight compounds, including 5,8-di (2,3-dihydroxy-3-methylbutoxy)-psoralen (**75**), heraclenol (**47**), IMP, isoimperatorin (**19**), phellopterin (**26**), scopoletin (**3**), byakangelicin (**41**) and ferulic acid (**292**) from *A. dahurica* root and evaluated their anti-microbial activities against *Bacillus subtilis*, *Escherichia coli*, *Cladosporium herbarum* and *Aspergillus candidus*. They found that these active constituents displayed good inhibitory effects against *Bacillus subtilis*, *Cladosporium herbarum* and *Aspergillus candidus* with minimun inhibitory concentration (MIC) of 62.5 μg/ml (Kwon et al., 1997). In bioassays of anti-microbial activity, the ethanol extract of *A. dahurica* root showed an inhibition radio of 40% against *Trypanosoma cruzi*, and the water extract of *A. dahurica* exhibited notable effect of anti-*Mycoplasma hominis* with a 50% minimun inhibitory concentration (MIC_50_) of 3.91 mg/ml, which could be used in treating *Mycoplasma hominis* infection (Schinella et al., 2002; Che et al., 2005). Moreover, the hexane extract of *A. dahurica* root was found to possess anti-microbial activity against *staphylococcus aureus*. From the hexane extract, an anti-microbial compound was isolated by bioassay-guided fractionation and identified as falcarindiol (**307**). In this study, falcarindiol inhibited the growth of *staphylococcal strains* with MICs ranged from 8 to 32 μg/ml (Lechner et al., 2004).

In addition to the anti-microbial activity, *A. dahurica* also presents significant antiviral effect. In recent years, coumarins from *A. dahurica* were reported to possess significant antiviral property. For example, *Lee et al.* found that four active furanocoumarins in the root of *A. dahurica*, including isoimperatorin (**19**), oxypeucedanin (**20**), oxypeucedanin hydrate (**21**) and IMP have significant antiviral activity against influenza A (H1N1 and H9N2) viruses. Among them, oxypeucedanin exhibit the most potent antiviral effect with an EC_50_ of 5.98 ± 0.71 and 4.52 ± 0.39, respectively. Further investigation showed that oxypeucedanin exerts anti-influenza A viruses property by inhibiting the virus infection-induced apoptosis and early stage of the viral replication cycle (Lee, B.W. et al., 2020). Besides, IMP (10, 25 and 50 μM, 30 min) was capable of inhibiting human immunodeficiency virus type 1 (HIV-1) replication in both T cells and HeLa cells infected by HIV-1 *via* the regulation of transcription factor specificity protein1 (Sp1) (Sancho et al., 2004). As many diseases occur due to the infection of bacteria and viruses. These studies suggested that the root of *A. dahurica* is a rich source of natural anti-microbial and antiviral agents that can prevent and treat some diseases.

### 5.6 Effects on the Cardiovascular System

Cardiovascular diseases are major contributor to global mortality and result in a huge socioeconomic burden. Many studies have decleared that *A. dahurica* extract and its active ingredients possess obvious protective role on cardiovascular system. Lee et al. (2015) first reported that 70% methanol extract of *A. dahurica* root (0.03–3.0 μg/ml) markedly relaxed calcium-induced vasocontraction of aortic rings in a concentration-dependent manner. In high-fat/high-fructose diet (HFFD)-fed rats, IMP at doses of 15 and 30 mg/kg b. w. for 4 weeks significantly reduced blood pressure and heart rate values, and alleviated changes in vascular morphology by regulating the expression of adiponectin receptor 1, endothelial nitric oxide synthase (eNOS) and p47^phox^ (Bunbupha et al., 2021). Meanwhile, IMP displayed a potent vasodilatation role by partially affecting the level of NO in phenylephrine-induced mouse thoracic aorta (IC_50_ = 12.2 ± 2.4 μmol/L) (Nie et al., 2009). He et al. (2007) found that IMP (1 μM–1 mM) might promote vasodilatation on arteries precontracted by agonists by regulating the calcium channel and competitively antagonizing 5-hydroxytryptamine (5-HT) recptors. Additionally, IMP could also attenuate pathological myocardial hypertrophy and cardiac fibrosis, inhibit transition to heart failure, and prevent cardiac myocyte protein synthesis and cell size induced by angiotensin II. The high dose of IMP (30 μM) was more effective than IMP (10 and 3 μM) and displayed concentration-dependently (Zhang et al., 2010). These scientific reports demonstrated that the root of *A. dahurica* and its active ingredients may control Ca^2+^ channel, modulate the expression of adiponectin receptor 1, eNOS and p47^phox^ to exert vasodilative and cardioprotective effects. IMP may be responsible for the effects of *A. dahurica* on cardiovascular system.

### 5.7 Effects on the Nervous System

IMP might largely contribute to the neuroprotective function of *A. dahurica* and possess significant properties on the nervous system, such as improving memory, antidepressive-like effect and anticonvulsant (Luszczki et al., 2009; Sigurdsson and Gudbjarnason, 2013; Cao et al., 2017). For example, pretreatment with IMP (5, 10 mg/kg b. w., 14 days) exhibited significant amelioration in the mice of LPS-induced poor memory retention by upregulating the level of brain derived neurotrophic factor (BDNF) and inhibiting oxidative stress and inflammation (Chowdhury et al., 2018). In middle cerebral artery occlusion (MCAO) rats, IMP at doses of 5 and 10 mg/kg b. w. reduced the infarct volume and increased the behavior ability. Moreover, IMP (0.612 and 2.56 μM) ameliorated the damage of neural cell lines (SH-SY5Y cells) by anti-apoptosis through increasing the expression of BDNF and phosphorylated-extracellular signal-regulated kinase (p-ERK) (Wang et al., 2013). In the maximal electroshock-induced seizure (MES) test, IMP at a dose of 50 mg/kg b. w. markedly enhanced the anticonvulsant activity of lamotrigine (LTG) in mice by reducing the 50% effective dose (ED_50_) value by 60% and increased the protective index from 4.90 to 8.96. The MES threshold for IMP administrated alone at 50 and 100 mg/kg were significantly increased by 38% and 68% at 30 min after its administration (Luszczki et al., 2007; Luszczki et al., 2008). In addition to IMP, some other compounds such as phellopterin (**26**) and scopoletin (**3**) also exhibit neuroprotective effects. Scopoletin (2, 10 and 50 mg/kg b. w.) administration for 2 weeks mitigated anxiety-like symptoms in complete Freund’s adjuvant (CFA)-induced mice by activating γ-aminobutyric acid (GABA_A_) receptors and phellopterin was reported to competitively bind to central nervous system benzodiazepine receptors with IC_50_ of 0.36 μM (Bergendorff et al., 1997; Luo et al., 2020). Alzheimer’s disease is a common neurodegenerative disease characterized by the formation of β-amyloid plaques and neurofibrillary tangles (Zhang et al., 2020). Marumoto et al. evaluated the inhibitory activities against β-secretase (BACE1) of five furanocoumarins from *A. dahurica* and found that IMP and byakangelicol (**34**) exhibit the most excellent properties with IC_50_ of 91.8 ± 7.5 and 104.9 ± 2.4 μM (Marumoto and Miyazawa, 2010), implying their potential for the treatment of Alzheimer’s disease. However, based on the present study, due to the complexities of the nervous system, IMP is limited to achieve desired therapeutic effects. The combination of IMP with other effective compounds may be a promising direction in clinical trials.

### 5.8 Hepatoprotective Activity

Several research publications reported the hepatoprotective activities of some active ingredients from *A. dahurica*. For example, Oh et al. isolated and identified six furocoumarins, including IMP, isoimperatorin (**19**), byakangelicol (**34**), oxypeucedanin (**20**), byakangelicin (**41**), and aviprin (**78**) from the methanol extract of *A. dahurica* root and validated their cytotoxic effect on tacrine-induced Hep G2 cells. Subsequently, IMP and byakangelicin displayed superior hepatoprotective activities with EC_50_ values of 36.6 ± 0.98 and 47.9 ± 4.6 μM, respectively. Byakangelicol and oxypeucedanin exhibited moderate hepatoprotective effects with EC_50_ values of 112.7 ± 5.35 and 286.7 ± 6.36 μM, respectively (Oh et al., 2002). Meanwhile, IMP at a dose of 100 mg/kg b. w. for 5 days was able to ameliorate acetaminophen overdose-induced acute liver injury in rats as evidenced by the reduced mortality, alanine aminotransferase (ALT) and aspartate aminotransferase (AST) in serum and centrilobular hepatic necrosis *via* stimulating the sirtuin 1 (SIRT1)-farnesoid X receptor (FXR) pathway (Gao et al., 2020). Furthermore, Oral administration of byakangelicin (100 mg/kg b. w., 4 weeks) in mice significantly improved carbon tetrachloride-induced liver fibrosis and damage by inhibiting the deposition of collagen and α-SMA, and decreasing the levels of ALT and AST in serum, which was more effective than that of silibinin. In 4-HNE–induced HepG2 cells, byakangelicin (20 and 40 μmol/L, 24 h) inhibited activation and proliferation of hepatic stellate cells, and prevented the apoptosis of hepatocyte through apoptosis signal regulating kinase-1 (ASK-1)/c-Jun N-terminal kinase (JNK) pathway (Li et al., 2020). The above mentioned results indicated that furocoumarins in *A. dahurica* exhibited obvious hepatoprotective activity and may be responsible for the hepatoprotective activity of *A. dahurica*. However, the molecular mechanism and clinical safety of some coumarins are not clear enough, which hinders the development of coumarins as hepatoprotective drugs. Future research should focus on the precise molecular mechanisms of furocoumarins in *A. dahurica*.

### 5.9 Effects on Skin Diseases

*A. dahurica* was extensively used as a traditional Chinese medicine in treating skin-associated diseases. In recent years, several studies revealed that *A. dahurica* has excellent activity on diabetes-induced skin ulcer (Guo et al., 2020; Chao et al., 2021; Hu et al., 2021). Guo et al. (2020) indicated that 10 days treatment with *A. dahurica* at 1.8 g/kg b. w. significantly promoted would healing and angiogenesis by activating phosphatidylinositide 3-kinase/protein kinase B (PI3K/AKT) and HIF-1α/platelet-derived growth factor-β (PDGF-β) pathways in db/db mice. Moreover, the 70% ethanol extract of *A. dahurica* root (2.5 mg/ml, 3 days) was reported to improve the adhesion of melanocytes to fibronectin and stimulate the migration of melanoctes to treat vitiligo (Zhang et al., 2005). Hwang et al. (2016) found that the root of *A. dahurica* methanol extract (50 μg/ml) and IMP (10, 20 and 40 μM, 2 days) markedly inhibited the insulin-like growth factor-1 (IGF-1)-induced sebum production by suppressing the phosphorylation of Akt and the expression of peroxisome proliferator-activated receptor-γ (PPAR-γ) and sterol response element-binding protein-1 (SREBP-1) in sebocytes, suggesting that they have the potential to be used in the treatment of acne. In addition, IMP and isoimperatorin (**19**) from *A. dahurica* root can inhibit melanogenesis by preventing tyrosinase synthesis in B16 melanoma cells and might have the potential to be exploit as a novel whitening agents in cosmetics (Cho et al., 2006). Collectively, the investigations mentioned above could partly support the claim about the traditional use of *A. dahurica* root for the treatment of skin diseases. The root of *A. dahurica* is commonly used to treat various skin diseases, such as scabies, carbuncle, sore and pruitus in China and other traditional medicinal systems in Asia, these indications may be promising for future clinical trials.

### 5.10 Other Activities

#### 5.10.1 Regulation of Lipid Metabolism

The 70% ethanol extract of *A. dahurica* root (800 mg/kg b. w., 4 weeks) significantly reduced the levels of TC and triglyceride (TG) in the livers of hyperlipidemia mice, as well as enhanced the activity of total hepatic lipolysis by upregulating the expression of PPARγ and lipid metabolism related genes-lipase member C (LIPC). Similarly, the levels of TC and TG were decreased by *A. dahurica* extract (400 μg/ml, 24 h) and IMP (20 μg/ml, 24 h) in 50% fetal bovine serum (FBS)-fed HepG2 cells (Lu et al., 2016). This study suggested the effect of *A. dahurica* root on the regulation of lipid metabolism and might be developed as a pharmaceutical product against fatty liver and hyperlipemia.

#### 5.10.2 Anti-Diabetic Activity

Phellopterin (**26**) isolated from ethyl acetate extract of *A. dahurica* root at doses of 1 and 2 mg/kg b. w. for 2 weeks significantly decreased the level of blood glucose, TC and TG in high-fat diets (HFD)/streptozotocin (STZ)-induced type Ⅱ diabetic mice. In 3T3-L1 preadipocytes, the ethyl acetate extract of *A. dahurica* (25, 50 and 100 μg/ml) and phellopterin (50 μg/ml, 9 days) induced adipocytes differentiation by increasing the expression of PPARγ, indicating the value of phellopterin for the development of anti-diabetic drugs through enhancing insulin sensitivity (Han et al., 2018).

#### 5.10.3 Immunoregulatory Activity

A latest study reported that ADP80-2, a water-soluble polysaccharide from *A. dahurica* root, at doses of 25, 50 and 100 μg/ml for 24 h promoted the phagocytosis of macrophage cells, the release of NO and the generation of cytokines (TNF-α, IL-6 and IL-1β). Moreover, ADP80-2 induced the production of immunoregulation-associated chemokines, including reactive oxygen species (ROS) and NO, in zebrafish embryos (Wang et al., 2021). Another study reported that ADP at doses of 10, 30 and 100 μg/ml activated the immune functions of dendritic cells by targeting toll-like receptor 4 (TLR4), MAPKs and NF-κB (Kim et al., 2013). These studies demonstrated that polysaccharides are mainly responsible for the immunomodulatory function in *A. dahurica*.

## 6 Pharmacokinetics

Pharmacokinetic studies on *A. dahurica* mainly focus on the coumarins. Ethanol extract of *A. dahurica* root was administrated orally at a dose of 4.5 g/kg b. w. to determine pharmacokinetic of nine coumarins, including IMP, isoimperatorin, oxypeucedanin hydrate, bergapton, oxypeucedanin, xanthotoxol, xanthotoxin, isopimpinellin and psoralen in the plasma of rats. The values of half-life time (t_1/2_) of these compounds were 2.4 ± 0.3, 2.2 ± 0.2, 4.8 ± 2.3, 1.8 ± 0.2, 2.4 ± 0.1, 4.8 ± 1.2, 4.5 ± 1.4, 2.8 ± 0.7 and 2.5 ± 0.7 h, respectively. Among these compounds, the values of maximum plasma concentration (C_max_) of IMP, isoimperatorin, oxypeucedanin hydrate and bergapten (1,017–2,900 ng/ml) were significantly higher than the other five compounds (21–138 ng/ml), which was consistent with their higher contents in *A. dahurica* (Chen et al., 2015). Xie and colleagues reported the pharmacokinetic profile oxypeucedanin hydrate and byakangelicin in the plasma of mongrel dogs after oral administration of *A. dahurica* ethanol extract (30 mg/kg b. w.). Oxypeucedanin hydrate reached a C_max_ of 4,154.09 ng/ml at 1.71 h (t_1/2_ 3.06 h), and byakangelicin reached a C_max_ of 1,474.72 ng/ml at 1.71 h (t_1/2_ 2.77 h) (Xie et al., 2007). Moreover, Hwang et al. (2017) used ultra-performance liquid chromatography-tandem mass spectrometry (UPLC/MS/MS) technology to identify the coumarins from the root of *A. dahurica*, including oxypeucedanin, IMP and isoimperatorin in the plasma of rats after oral administration (0.5 g/kg b. w.). They found that all the three compounds had rapid oral absorption with the time to reach the peak concentration (T_max_) of 40–75 min. Oxypeucedanin reached a C_max_ of 38.5 ng/ml at 43.2 min (t_1/2_ 78.1 min), IMP reached a C_max_ of 94.5 ng/ml at 54.0 min (t_1/2_ 59.5 min), and isoimperatorin reached a C_max_ of 72.1 ng/ml at 72.0 min (t_1/2_ 63.8 min). Furthermore, it is worth noting that *A. dahurica* has also been found to affect metabolism of some drugs. The water extract of *A. dahurica* root (oral dose of 1 mg/kg b. w.) significantly increased the area under the concentration–time curve (AUC), t_1/2_ and plasma clearance (CL) by 2.5, 2.3 and 0.45 times, respectively after tolbutamide was administrated intravenously in rats, suggesting that *A. dahurica* delayed elimination of tolbutamide. Moreover, treatment with the root of *A. dahurica* markedly increased the C_max_ by 4 times after oral administration of diazepam in rats, indicating that the first-pass effect of the drug was attenuated. Meanwhile, *A. dahurica* could also increase the duration of rotarod disruption of diazepam. Mechanistically, *A. dahurica* interfered the metabolism of tolbutamide and diazepam by inhibiting the activity of cytochrome P450 (Ishihara et al., 2000). These findings implied the potential of *A. dahurica* to be adjuvant therapy of drugs in some specific diseases.

In conclusion, these results indicated that the pharmacokinetic parameters of single compound and the extract of *A. dahurica* after oral administration may vary due to dosage form and composition. On the whole, investigations on pharmacokinetics for *A. dahurica* are relatively limited. Future work should focus more on the pharmacokinetics of *A. dahurica* in order to better evaluate its clinical efficacy.

## 7 Quality Control

It is well known that quality control of herb medicine plays an essential role in ensuring their safety and efficiency. According to the Chinese pharmacopoeia (the 2020 edition), the content of IMP in the root of *A. dahurica* must be no less than 0.080%, and the total ash should not exceed 6.0%, which is consistent with the European Pharmacopoeia (the 10th edition). Meanwhile, the Chinese Pharmacopoeia stipulates that the moisture content in *A. dahurica* should not more than 14.0%, and the European Pharmacopoeia states that the moisture content should less than 12.0%. In addition, according to the description in the Japanese Pharmacopoeia (the 18th edition) and the Korean Pharmacopoeia (the 8th edition), the total ash content, acid-insoluble ash content and ethanol extract should less than 7.0%, 2.5%, more than 25%, respectively. However, the inherent quality of medicinal plants may be affected by geographical conditions, harvest time, cultivation techniques and many other factors (Cheng et al., 2019). For instance, Yang et al. (2020) found that the contents of IMP in different regions of China were variable. Among them, the highest content of IMP was 0.392% in Yangjiaying, Hebei, followed by 0.363% in Xiaoying, Hebei, and the lowest content was 0.093% in Mengzhou, Henan. In addition, TCM usually exert its curative effects through the synergistic effect of multiple components, and it is insufficient to determine the quality of *A. dahurica* by relying on only a single component for quality control. With the development of analytical techniques, the multi-component determination has been prevalently used in the comprehensive quality control of compounds isolated from *A. dahurica*. A total of 21 courmarins: IMP, byakangelicin, oxypeucedanin, bergapten, cnidilin, osthole, isoimperatorin, scopoletin, xanthotoxol, xanthotoxin, psoralen, isoimpinellin, andafocoumarins A, B, C, D, E, F, G, H, J and some volatile oils have been quantified by different analytical tools. The quantitative analysis of the compounds isolated from *A. dahurica* is listed in Table 6. Besides, the fingerprint analysis has also been apply to the quality assessment of *A. dahurica*. Kang et al. (2008) found that the 13 batches of *A. dahurica* root from different regions had similar high performance liquid chromatography (HPLC) fingerprints and indicated that fingerprint method could be used for the quality control of *A. dahurica*. The fingerprint method can also detect the mixing of *A. dahurica* and the root of other *Angelica* species and other putative contaminations. Wang Y. J. et al. (2020) indicated that the peak shapes of *A. dahurica* and the roots of other *Angelica* species, including *A. pubescens* and *A. sinensis* are quite different and can be distinguished by HPLC fingerprints through different chemical components. Moreover, the mixing of *A. dahurica* and the root of other *Angelica* species and other putative contaminations can be detected according to the different characteristic peaks from HPLC fingerprints.

**TABLE 6:** Quantitative analysis for the quality control of *A. dahurica* root.

Analytes	Methods	Results	References
IMP, byakangelicin and oxypeucedanin	^1^H-qNMR	The contents were 0.093%–0.392%, 0.117%–0.315% and 0.173%–0.353% for IMP, byakangelicin and oxypeucedanin, respectively, and they were in different batches of *A. dahurica*	Yang et al. (2020)
Volatile oils	GC-MS	22 volatile oils were identified from *A. dahurica*, main including hexadecanoic acid, ethyl ester (7.32%), α-pinene (6.25%), dodecyl alcohol (13.71%), 1-pentadecanol (6.08%) and elemene (7.54%)	Sun et al. (2017)
Coumarins	LC-MS/MS	9 furanocoumarins have been quantified in the injection of 20 batches of *A. dahurica* with contents of 3.60–333.33, 0.86–77.48, 1.20–41.74, 3.35–146.84, 1.38–44.51, 3.68–71.82, 21.83–411.03, 9.28–218.73 and 4.11–303.58 μg/g for andafocoumarins A, B, C, D, E, F, G, H, and J	Lei Zhang et al. (2017)
Volatile oils	GC-MS	38 compounds were identified from the essential oils of *A. dahurica* roots, mainly including α-pinene (44.91%), myrcene (8.72%), terpinen-4-ol (8.01%), cryptone (6.67%), 1-dodecanol (6.43%) and sabinene (3.42%)	Dongying Wang et al. (2020)
Coumarins	HPLC–ESIMS/MS	11 coumarins have been quantified in the injection of 12 batches of *A. dahurica* with contents of 26.1–396.6, 4.2–67.9, 23.1–99.0, 3.1–49.0, 2.6–14.8, 25.6–217.3, 81.3–2079.2, 355.1–1,418.6, 312.5–894.8, 0.3–2.2, 364.4–900.4 and 1,327.7–5892.9 μg/g for scopoletin, xanthotoxol, xanthotoxin, psoralen, isoimpinellin, bergapten, oxypeucedanin, IMP, cnidilin, osthole and isoimperatorin	Zheng et al. (2010)
Bergapten, IMP, cnidilin, osthole and isoimperatorin	HPLC	The contents were 181.2–1,152.9, 479.8–1889.3, 321.3–903.1, 7.3–37.7 and 329.7–723.2 μg/g for bergapten, IMP, cnidilin, osthole and isoimperatorin, respectively, and they were 21 batches of *A. dahurica*	Wang et al. (2007)

## 8 Safety

As a common used medicinal and edible substance, *A. dahurica* plays an important role in the health of human body. The toxicity investigations on the safety for *A. dahurica* are relatively lacking, although this plant exhibits extensive pharmacological activities. Zheng et al. (2012) compared the acute toxicity of sulphur fumigated and non-sulphur-fumigated *A. dahurica* extracts and indicated that both of them belong to non-toxic grade. The 50% lethal dose (LD_50_) of non-sulphur-fumigated *A. dahurica* extracts in Kunming mice was 55.5169 g/kg, while the LD_50_ of sulphur-fumigated *A. dahurica* extracts in Kunming mice was 89.4420 g/kg, suggesting the safety of *A. dahurica* and sulphur fumigation could reduce the toxicity of *A. dahurica*.

## 9 Conclusion and Future Perspectives

In this review, we summarized the traditional uses, phytochemistry and pharmacology activities of *A. dahurica* according to ancient classics and modern researches, and it will provide a new insight for future exploration of *A. dahurica*. The root of *A. dahurica* has been widely used to treat cold fever, headache, toothache and cold-damp pain in ancient and modern China. Meanwhile, the root of *A. dahurica* has a predominant therapeutic effect in diseases such as abnormal leucorrhea, sore, as well as skin ulcer. Interestingly, *A. dahurica* root exerts dual functions as medicine and food, which has been widely used as condiment or healthcare product. Up to now, more than 300 compounds have been isolated and identified from *A. dahurica*. Among these constituents, coumarins and volatile oils represent the main active ingredients and IMP (22) is the most principal and representative compound of *A. dahurica*. It is expected that more compounds of these categories will be discovered in the future studies. Moreover, researches have shown that both crude extracts and active components of *A. dahurica* possess a wide range of pharmacological activities, including anti-inflammation, anti-tumor, anti-oxidation, analgesic activity, antiviral and anti-microbial effects, effects on the cardiovascular system, neuroprotective function, hepatoprotective activity, effects on skin diseases and so on. These modern pharmacological studies supported most traditional uses of *A. dahurica* root as folk medicine. However, gaps still exist in the systematic research on *A. dahurica*.

Firstly, the chemical constituents and pharmacological studies of the aerial part are limited, although the roots of *A. dahurica* have been studied extensively in recent decades. Current studies of *A. dahurica* most focused on the crude extracts and some coumarins such as IMP, byakangelicin, phellopterin and scopoletin, but these investigations are insufficient. Studies have shown that the aerial part of *A. dahurica* also has certain pharmacological activities, such as anti-oxidation (Lee and Woo, 2011), and thus might have medicinal relevance for some aging-related diseases. Therefore, more extensive studies of other compositions and other parts of *A. dahurica* are necessary. Secondly, many pharmacological studies on the crude extracts or active components are not in-depth enough. These pharmacological activities need to be further confirmed by animal experiments *in vivo* and combined with clinical applications. This direction will provide a solid basis for developing novel drug-lead compounds in the future study. For example, the minimum effect dose (MED) of IMP in two kidney one clip renovascular hypertensive rats (2K1C-RHR) was 6.25 mg/kg, and it exhibited obvious hypotensive effect after continuous administration for 2 weeks. The proposed clinical dose of IMP is 100 mg/d per person, that is, 1.67 mg/kg. Moreover, the LD_50_ of IMP in rats was 3188.7 mg/kg, indicating that IMP has a wide safety range and a great possibility of clinical application (Zhu et al., 2013).

Thirdly, most studies on the pharmacological activities of *A. dahurica* concentrated on uncharacterized crude extracts, and this makes it difficult to clarify the connections between bioactivities and isolated compounds. Furhter systematic pharmacological studies of the compounds isolated from *A. dahurica* are quite considerable. Additionally, the exact mechanisms of many pharmacological activities, such as anti-oxidant and antiviral activities of the crude extract or compounds from *A. dahurica* remain unclear; thus, further studies to better reveal the precise molecular mechanisms of the pharmacological activities of this herb seem to be necessary.

Fourthly, there were multiple processing methods of *A. dahurica* root in ancient China, such as stir-baking with *Polygonati Rhizoma*, immersing into wine and immersing into rice. Different processing methods may affect the chemical constituents and pharmacological activities of *A. dahurica* root, resulting in different clinical applications, but there are few studies on the influences of processing methods of *A. dahurica* root. Hence, investigations on the processing methods may be one of the main directions of *A. dahurica* root in the future researches.

Finally, *A. dahurica* root is usually used prescribed with other traditional herbs, such as *Atractylodes lancea* and *Xanthium sibiricum* to treat specific diseases. However, only a few studies to reveal the effects of synergy or antagonism have been reported. Therefore, the roles on drug-interaction between certain herbs and *A. dahurica* seem to be a new direction that worth further exploration.

In conclusion, the root of *A. dahurica* is an important edible medicinal herb with extensive pharmacological activities and great values in medicine and food. However, more in-depth and comprehensive studies on clinical utility are needed to determine its safety and availability. Until now, multiple compounds have been discovered in *A. dahurica*, but what we have done is far from enough. Moreover, the precise molecular mechanisms of these active ingredients in some diseases still worth further study. Consequently, systematic studies on phytochemistry and bioactivities of *A. dahurica* will undoubtedly be the key direction of future research. This review should provide an important reference for the development and application of *A. dahurica*.

## Abbreviations

ADP, *Angelica dahurica* polysaccharide; ALT, aminotransferase; AST, aspartate aminotransferase; BDNF, brain derived neurotrophic factor; b.w., body weight; CAD, coumarins of *Angelica dahurica*; C_max_, maximum concentration; COX-2, cyclooxygenase-2; CYP, cytochrome P450; DPPH, 1,1-diphenyl-2-picrylhydrazyl; EC_50_, 50% effective concentration; IC_50_, 50% inhibitory concentration; HPLC, high performance liquid chromatography; IL-1β, interleukin-1β; IMP, imperatorin; iNOS, inducible nitric oxide synthase; LD_50_, 50% lethal dose; LDL-C, low density lipoprotein cholesterol; LPS, lipopolysaccharide; MAPK, mitogen-activated protein kinase; MES, maximal electroshock-induced seizure; MIC, minimun inhibitory concentration; NF-κ B, nuclear factor-κB; NO, nitric oxide; PPARγ, peroxisome proliferator-activated receptor γ; TC, total cholesterol; TCM, traditional Chinese medicine; TNF-α, tumor necrosis factor-α; t_1/2_, half-life period; TRPV1, Transient receptor potential vanilloid type 1.

## References

[B1] Babu S. Jayaraman S. (2020). An Update on β-sitosterol: A Potential Herbal Nutraceutical for Diabetic Management. Biomed. Pharmacother. 131, 110702. 10.1016/j.biopha.2020.110702 32882583

[B2] Baek N. I. Ahn E. M. Kim H. Y. Park Y. D. (2000). Furanocoumarins from the Root of Angelica Dahurica. Arch. Pharm. Res. 23 (5), 467–470. 10.1007/bf02976574 11059825

[B3] Bai Y. Li D. Zhou T. Qin N. Li Z. Yu Z. (2016). Coumarins from the Roots of Angelica Dahurica with Antioxidant and Antiproliferative Activities. J. Funct. Foods 20, 453–462. 10.1016/j.jff.2015.11.018

[B128] Banikazemi Z. Mirazimi S. M. Dashti F. Mazandaranian M. R. Akbari M. Morshedi K. (2021). Coumarins and Gastrointestinal Cancer: A New Therapeutic Option?. Front. Oncol. 11, 752784. 10.3389/fonc.2021.752784 34707995 PMC8542999

[B4] Bergendorff O. Dekermendjian K. Nielsen M. Shan R. Witt R. Ai J. (1997). Furanocoumarins with Affinity to Brain Benzodiazepine Receptors *In Vitro* . Phytochemistry 44 (6), 1121–1124. 10.1016/s0031-9422(96)00703-0 9055449

[B5] Bunbupha S. Prasarttong P. Poasakate A. Maneesai P. Pakdeechote P. (2021). Imperatorin Alleviates Metabolic and Vascular Alterations in High-Fat/high-Fructose Diet-Fed Rats by Modulating Adiponectin Receptor 1, eNOS, and P47phox Expression. Eur. J. Pharmacol. 899, 174010. 10.1016/j.ejphar.2021.174010 33711309

[B6] Cao Y. Liu J. Wang Q. Liu M. Cheng Y. Zhang X. (2017). Antidepressive-like Effect of Imperatorin from Angelica Dahurica in Prenatally Stressed Offspring Rats through 5-hydroxytryptamine System. Neuroreport 28 (8), 426–433. 10.1097/wnr.0000000000000778 28383321 PMC5639996

[B7] Cascaes M. M. Carneiro O. D. S. Nascimento L. D. D. de Moraes Â. A. B. de Oliveira M. S. Cruz J. N. (2021). Essential Oils from Annonaceae Species from Brazil: A Systematic Review of Their Phytochemistry, and Biological Activities. Int. J. Mol. Sci. 22 (22), 12140. 10.3390/ijms222212140 34830022 PMC8623146

[B8] Chang Y. Y. Tsai Y. T. Lai J. N. Yeh C. H. Lin S. K. (2014). The Traditional Chinese Medicine Prescription Patterns for Migraine Patients in Taiwan: A Population-Based Study. J. Ethnopharmacol. 151 (3), 1209–1217. 10.1016/j.jep.2013.12.040 24389028

[B9] Chao Y.-H. Yang W.-T. Li M.-C. Yang F.-L. Lee R.-P. (2021). Angelica Dahurica and Rheum Officinale Facilitated Diabetic Wound Healing by Elevating Vascular Endothelial Growth Factor. Am. J. Chin. Med. 49 (6), 1515–1533. 10.1142/s0192415x21500713 34224339

[B10] Che Y. M. Mao S. H. Jiao W. L. Fu Z. Y. (2005). Susceptibilities of Mycoplasma Hominis to Herbs. Am. J. Chin. Med. 33 (2), 191–196. 10.1142/s0192415x05002862 15974478

[B11] Chen L. Jian Y. Wei N. Yuan M. Zhuang X. Li H. (2015). Separation and Simultaneous Quantification of Nine Furanocoumarins from Radix Angelicae Dahuricae Using Liquid Chromatography with Tandem Mass Spectrometry for Bioavailability Determination in Rats. J. Sep. Sci. 38 (24), 4216–4224. 10.1002/jssc.201500840 26496866

[B12] Chen L. Xu W. Yang Q. Zhang H. Wan L. Xin B. (2020). Imperatorin Alleviates Cancer Cachexia and Prevents Muscle Wasting via Directly Inhibiting STAT3. Pharmacol. Res. 158, 104871. 10.1016/j.phrs.2020.104871 32413482

[B13] Chen W. Wang G. Mei K. Zhu J. (2021). Coumarins from Angelica Dahurica and Their Antitumor Activities in Human MG-63 Osteosarcoma Cells. Rec. Nat. Prod. 15 (5), 356–362. 10.25135/rnp.225.21.01.1935

[B14] Chen X. Sun W. Gianaris N. G. Riley A. M. Cummins T. R. Fehrenbacher J. C. (2014). Furanocoumarins Are a Novel Class of Modulators for the Transient Receptor Potential Vanilloid Type 1 (TRPV1) Channel. J. Biol. Chem. 289 (14), 9600–9610. 10.1074/jbc.M113.536862 24569998 PMC3975010

[B15] Chen Y. She Y. Kaur R. Guo N. Zhang X. Zhang R. (2019). Is Plant Sterols a Good Strategy to Lower Cholesterol? J. Oleo. Sci. 68 (9), 811–816. 10.5650/jos.ess19116 31413246

[B16] Cheng J. Dang P. P. Zhao Z. Yuan L. C. Zhou Z. H. Wolf D. (2019). An Assessment of the Chinese Medicinal Dendrobium Industry: Supply, Demand and Sustainability. J. Ethnopharmacol. 229, 81–88. 10.1016/j.jep.2018.09.001 30266420

[B17] Chinese Pharmacopoeia Commission (2020). Chinese Pharmacopeia. Beijing: China Medical Science Press, 109.

[B18] Cho Y. H. Kim J. H. Park S. M. Lee B. C. Pyo H. B. Park H. D. (2006). New Cosmetic Agents for Skin Whitening from Angelica Dahurica. J. Cosmet. Sci. 57 (1), 11–21. 16676120

[B19] Choi S. Y. Ahn E. M. Song M. C. Kim D. W. Kang J. H. Kwon O. S. (2005). *In Vitro* GABA-transaminase Inhibitory Compounds from the Root of Angelica Dahurica. Phytother. Res. 19 (10), 839–845. 10.1002/ptr.1424 16261512

[B20] Choochuay K. Chunhacha P. Pongrakhananon V. Luechapudiporn R. Chanvorachote P. (2013). Imperatorin Sensitizes Anoikis and Inhibits Anchorage-independent Growth of Lung Cancer Cells. J. Nat. Med. 67 (3), 599–606. 10.1007/s11418-012-0719-y 23108812

[B21] Chowdhury A. A. Gawali N. B. Shinde P. Munshi R. Juvekar A. R. (2018). Imperatorin Ameliorates Lipopolysaccharide Induced Memory Deficit by Mitigating Proinflammatory Cytokines, Oxidative Stress and Modulating Brain-Derived Neurotropic Factor. Cytokine 110, 78–86. 10.1016/j.cyto.2018.04.018 29705395

[B22] Chung I. M. Kim E. H. Lee J. H. Lee Y. C. Moon H. I. (2012). Immunotoxicity Activity from Various Essential Oils of Angelica Genus from South Korea against *Aedes aegypti* L. Immunopharmacol. Immunotoxicol. 34 (1), 42–45. 10.3109/08923973.2011.572891 21506693

[B23] Deng G. G. Gui Z. J. Yang X. W. (2015a). Chemical Constituents from Polarity Part in Roots of Angelica Dahurica Var. Formosana Cv. Chuanbaizhi. Zhongguo Zhong Yao Za Zhi 40 (19), 3805–3810. doi:10.4268 /cjcmm20151920 26975106

[B24] Deng G. G. Wei W. Yang X. W. Zhang Y. B. Xu W. Gong N. B. (2015b). New Coumarins from the Roots of Angelica Dahurica Var. Formosana Cv. Chuanbaizhi and Their Inhibition on NO Production in LPS-Activated RAW264.7 Cells. Fitoterapia 101, 194–200. 10.1016/j.fitote.2015.01.016 25647326

[B25] Deng M. Xie L. Zhong L. Liao Y. Liu L. Li X. (2020). Imperatorin: A Review of its Pharmacology, Toxicity and Pharmacokinetics. Eur. J. Pharmacol. 879, 173124. 10.1016/j.ejphar.2020.173124 32339515

[B26] Dong X. D. Liu Y. N. Zhao Y. Liu A. J. Ji H. Y. Yu J. (2021). Structural Characterization of a Water-Soluble Polysaccharide from Angelica Dahurica and its Antitumor Activity in H22 Tumor-Bearing Mice. Int. J. Biol. Macromol. 193, 219–227. 10.1016/j.ijbiomac.2021.10.110 34688677

[B27] Flora of China Editorial Committee (2006). Flora of China. Beijing, China: Science Press.

[B28] Fujiwara H. Yokoi T. Tani S. Saiki Y. Kato A. (1980). Studies on Constituents of Angelicae Dahuricae Radix. I. On a New Furocoumarin Derivative (Author's Transl). Yakugaku. Zasshi. 100 (12), 1258–1261. 10.1248/yakushi1947.100.12_1258 7252783

[B29] Gao Z. Zhang J. Wei L. Yang X. Zhang Y. Cheng B. (2020). The Protective Effects of Imperatorin on Acetaminophen Overdose-Induced Acute Liver Injury. Oxid. Med. Cell. Longev. 2020, 8026838. 10.1155/2020/8026838 32454943 PMC7243017

[B30] Guo J. Hu Z. Yan F. Lei S. Li T. Li X. (2020). Angelica Dahurica Promoted Angiogenesis and Accelerated Wound Healing in Db/db Mice via the HIF-1α/pdgf-β Signaling Pathway. Free Radic. Biol. Med. 160, 447–457. 10.1016/j.freeradbiomed.2020.08.015 32853721

[B31] Guo J. Chen D. Zhu C. Tang Z. X. (2019). Analgesic Effect and Analgesic Mechanism of Angelica Dahurica Extracts. J. Guangxi. Norm. Univ. 37 (4), 103–110. 10.16088/j.issn.1001-6600.2019.04.013

[B32] Han H. S. Jeon H. Kang S. C. (2018). Phellopterin Isolated from Angelica Dahurica Reduces Blood Glucose Level in Diabetic Mice. Heliyon 4 (3), e00577. 10.1016/j.heliyon.2018.e00577 29862342 PMC5968131

[B33] He J. Y. Zhang W. He L. C. Cao Y. X. (2007). Imperatorin Induces Vasodilatation Possibly via Inhibiting Voltage Dependent Calcium Channel and Receptor-Mediated Ca2+ Influx and Release. Eur. J. Pharmacol. 573 (1-3), 170–175. 10.1016/j.ejphar.2007.06.043 17662269

[B34] Hu D. Guo J. Li T. Zhao M. Zou T. Song H. (2019). Comparison and Identification of the Aroma-Active Compounds in the Root of Angelica Dahurica. Molecules 24 (23), 4352. 10.3390/molecules24234352 PMC6930666 31795226

[B35] Hu Y. Lei S. Yan Z. Hu Z. Guo J. Guo H. (2021). Angelica Dahurica Regulated the Polarization of Macrophages and Accelerated Wound Healing in Diabetes: A Network Pharmacology Study and *In Vivo* Experimental Validation. Front. Pharmacol. 12, 678713. 10.3389/fphar.2021.678713 34234674 PMC8256266

[B36] Hua J. M. Moon T. C. Hong T. G. Park K. M. Son J. K. Chang H. W. (2008). 5-Methoxy-8-(2-hydroxy-3-buthoxy-3-methylbutyloxy)-psoralen Isolated from Angelica Dahurica Inhibits Cyclooxygenase-2 and 5-Lipoxygenase in Mouse Bone Marrow-Derived Mast Cells. Arch. Pharm. Res. 31 (5), 617–621. 10.1007/s12272-001-1202-9 18481018

[B37] Huang R. Liu Y. Chen J. Lu Z. Wang J. He W. (2022). Limited Genetic Diversity and High Differentiation in Angelica Dahurica Resulted from Domestication: Insights to Breeding and Conservation. Bmc. Plant. Biol. 22 (1), 141. 10.1186/s12870-022-03545-5 35331143 PMC8953045

[B38] Hwang Y. H. Yang H. J. Ma J. Y. (2017). Simultaneous Determination of Three Furanocoumarins by UPLC/MS/MS: Application to Pharmacokinetic Study of Angelica Dahurica Radix after Oral Administration to Normal and Experimental Colitis-Induced Rats. Molecules 22 (3), 416. 10.3390/molecules22030416 PMC6155430 28272365

[B39] Hwang Y. L. Im M. Lee M. H. Roh S. S. Choi B. W. Kim S. J. (2016). Inhibitory Effect of Imperatorin on Insulin-like Growth Factor-1-Induced Sebum Production in Human Sebocytes Cultured *In Vitro* . Life. Sci. 144, 49–53. 10.1016/j.lfs.2015.11.027 26631504

[B40] Hwangbo H. Choi E. O. Kim M. Y. Kwon D. H. Ji S. Y. Lee H. (2020). Suppression of Tumor Growth and Metastasis by Ethanol Extract of Angelica Dahurica Radix in Murine Melanoma B16F10 Cells. Biosci. Trends. 14 (1), 23–34. 10.5582/bst.2019.01230 32092745

[B41] Iftinca M. Defaye M. Altier C. (2021). TRPV1-Targeted Drugs in Development for Human Pain Conditions. Drugs 81 (1), 7–27. 10.1007/s40265-020-01429-2 33165872

[B42] Ishihara K. Kushida H. Yuzurihara M. Wakui Y. Yanagisawa T. Kamei H. (2000). Interaction of Drugs and Chinese Herbs: Pharmacokinetic Changes of Tolbutamide and Diazepam Caused by Extract of Angelica Dahurica. J. Pharm. Pharmacol. 52 (8), 1023–1029. 10.1211/0022357001774750 11007075

[B43] Jia M. Li Y. Xin H. L. Hou T. T. Zhang N. D. Xu H. T. (2016). Estrogenic Activity of Osthole and Imperatorin in MCF-7 Cells and Their Osteoblastic Effects in Saos-2 Cells. Chin. J. Nat. MedJ. Nat. Med. 14 (6), 413–420. 10.1016/S1875-5364(16)30037-1 27473958

[B44] Jia X. Feng X. Zhao X. Dong Y. Zhao Y. Sun H. (2008a). Two New Linear Furanocoumarin Glycosides from Angelica Dahurica. Chem. Nat. Compd. 44 (2), 166–168. 10.1007/s10600-008-9004-4

[B45] Jia X. Zhao X. Wang M. Chen Y. Dong Y. Feng X. (2008b). Two New Coumarin Biosides from Angelica Dahurica. Chem. Nat. Compd. 44 (6), 692–695. 10.1007/s10600-009-9199-z

[B46] Kang J. Zhou L. Sun J. Han J. Guo D. A. (2008). Chromatographic Fingerprint Analysis and Characterization of Furocoumarins in the Roots of Angelica Dahurica by HPLC/DAD/ESI-MSn Technique. J. Pharm. Biomed. Anal. 47 (4-5), 778–785. 10.1016/j.jpba.2008.03.010 18436412

[B47] Kang U. Han A. R. So Y. Jin C. H. Ryu S. M. Lee D. (2019). Furanocoumarins from the Roots of Angelica Dahurica with Inhibitory Activity against Intracellular Reactive Oxygen Species Accumulation. J. Nat. Prod. 82 (9), 2601–2607. 10.1021/acs.patprod.9b0054710.1021/acs.jnatprod.9b00547 31464439

[B48] Khodarahmi G. Asadi P. Hassanzadeh F. Khodarahmi E. (2015). Benzofuran as a Promising Scaffold for the Synthesis of Antimicrobial and Antibreast Cancer Agents: A Review. J. Res. Med. Sci. 20 (11), 1094–1104. 10.4103/1735-1995.172835 26941815 PMC4755098

[B49] Kim H. S. Shin B. R. Lee H. K. Park Y. S. Liu Q. Kim S. Y. (2013). Dendritic Cell Activation by Polysaccharide Isolated from Angelica Dahurica. Food. Chem. Toxicol. 55, 241–247. 10.1016/j.fct.2012.12.007 23246459

[B50] Kimura Y. Okuda H. (1997). Histamine-release Effectors from Angelica Dahurica Var. Dahurica Root. J. Nat. Prod. 60 (3), 249–251. 10.1021/np960407a 9157191

[B51] Kwon Y. S. Kobayashi A. Kajiyama S. Kawazu K. Kanzaki H. Kim C. M. (1997). Antimicrobial Constituents of Angelica Dahurica Roots. Phytochemistry 44 (5), 887–889. 10.1016/s0031-9422(96)00634-6 9115693

[B52] Kwon Y. S. Shin S. J. Kim M. J. Kim C. M. (2002). A New Coumarin from the Stem of Angelica Dahurica. Arch. Pharm. Res. 25 (1), 53–56. 10.1007/bf02975261 11885692

[B53] Lechner D. Stavri M. Oluwatuyi M. Pereda-Miranda R. Gibbons S. (2004). The Anti-staphylococcal Activity of Angelica Dahurica (Bai Zhi). Phytochemistry 65 (3), 331–335. 10.1016/j.phytochem.2003.11.010 14751304

[B54] Lee B. W. Ha T. K. Q. Cho H. M. An J. P. Kim S. K. Kim C. S. (2020a). Antiviral Activity of Furanocoumarins Isolated from Angelica Dahurica against Influenza a Viruses H1N1 and H9N2. J. Ethnopharmacol. 259, 112945. 10.1016/j.jep.2020.112945 32389854

[B55] Lee H. J. Lee H. Kim M. H. Choi Y. Y. Ahn K. S. Um J. Y. (2017). Angelica Dahurica Ameliorates the Inflammation of Gingival Tissue via Regulation of Pro-inflammatory Mediators in Experimental Model for Periodontitis. J. Ethnopharmacol. 205, 16–21. 10.1016/j.jep.2017.04.018 28455165

[B56] Lee K. Shin M. S. Ham I. Choi H. Y. (2015). Investigation of the Mechanisms of Angelica Dahurica Root Extract-Induced Vasorelaxation in Isolated Rat Aortic Rings. BMC Complement. Altern. Med. 15, 395. 10.1186/s12906-015-0889-8 26520575 PMC4628382

[B57] Lee M. Y. Seo C. S. Lee J. A. Lee N. H. Kim J. H. Ha H. (2011). Anti-asthmatic Effects of Angelica Dahurica against Ovalbumin-Induced Airway Inflammation via Upregulation of Heme Oxygenase-1. Food. Chem. Toxicol. 49 (4), 829–837. 10.1016/j.fct.2010.12.004 21146576

[B58] Lee S. H. Han A.-R. Kang U. Kim J.-B. Seo E. K. Jung C.-H. (2020b). Inhibitory Effects of Furanocoumarins from the Roots ofAngelica Dahuricaon Ionizing Radiation-Induced Migration of A549 Human Non-small Cell Lung Cancer Cells. Nat. Product. Commun. 15 (4), 1934578X2091503. 10.1177/1934578x20915036

[B59] Lee Y.-S. Kim N.-W. (2011). Antioxidant Activity and Irritation Test of Extracts Obtained from Angelica Dahurica. Jfn 16 (1), 8–11. 10.3746/jfn.2011.16.1.008

[B60] Li B. Zhang X. Wang J. Zhang L. Gao B. Shi S. (2014). Simultaneous Characterisation of Fifty Coumarins from the Roots of Angelica Dahurica by Off-Line Two-Dimensional High-Performance Liquid Chromatography Coupled with Electrospray Ionisation Tandem Mass Spectrometry. Phytochem. Anal. 25 (3), 229–240. 10.1002/pca.2496 24481589

[B61] Li D. Wu L. (2017). Coumarins from the Roots of Angelica Dahurica Cause Anti-allergic Inflammation. Exp. Ther. Med. 14 (1), 874–880. 10.3892/etm.2017.4569 28673013 PMC5488689

[B62] Li X. Shao S. Li H. Bi Z. Zhang S. Wei Y. (2020). Byakangelicin Protects against Carbon Tetrachloride-Induced Liver Injury and Fibrosis in Mice. J. Cell. Mol. Med. 24 (15), 8623–8635. 10.1111/jcmm.15493 32643868 PMC7412405

[B63] Liang W. H. Chang T. W. Charng Y. C. (2018). Effects of Drying Methods on Contents of Bioactive Compounds and Antioxidant Activities of Angelica Dahurica. Food. Sci. Biotechnol. 27 (4), 1085–1092. 10.1007/s10068-018-0359-4 30263838 PMC6085254

[B64] Lin M. Zhang W. Su J. (2016). Toxic Polyacetylenes in the Genus Bupleurum (Apiaceae) - Distribution, Toxicity, Molecular Mechanism and Analysis. J. Ethnopharmacol. 193, 566–573. 10.1016/j.jep.2016.09.052 27693772

[B65] Liu L. Shan L. P. Xue M. Y. Lu J. F. Hu Y. Liu G. L. (2021). Potential Application of Antiviral Coumarin in Aquaculture against IHNV Infection by Reducing Viral Adhesion to the Epithelial Cell Surface. Antivir. Res. 195, 105192. 10.1016/j.antiviral.2021.105192 34687821

[B66] Lu X. Yuan Z. Y. Yan X. J. Lei F. Jiang J. F. Yu X. (2016). Effects of Angelica Dahurica on Obesity and Fatty Liver in Mice. Chin. J. Nat. Med. 14 (9), 641–652. 10.1016/s1875-5364(16)30076-0 27667509

[B67] Luo L. Sun T. Yang L. Liu A. Liu Q. Q. Tian Q. Q. (2020). Scopoletin Ameliorates Anxiety-like Behaviors in Complete Freund's Adjuvant-Induced Mouse Model. Mol. Brain. 13 (1), 15. 10.1186/s13041-020-0560-2 32019580 PMC7001522

[B68] Luszczki J. J. Glowniak K. Czuczwar S. J. (2007). Time-course and Dose-Response Relationships of Imperatorin in the Mouse Maximal Electroshock Seizure Threshold Model. Neurosci. Res. 59 (1), 18–22. 10.1016/j.neures.2007.05.004 17602770

[B69] Luszczki J. J. Wojda E. Andres-Mach M. Cisowski W. Glensk M. Glowniak K. (2009). Anticonvulsant and Acute Neurotoxic Effects of Imperatorin, Osthole and Valproate in the Maximal Electroshock Seizure and Chimney Tests in Mice: A Comparative Study. Epilepsy. Res. 85 (2-3), 293–299. 10.1016/j.eplepsyres.2009.03.027 19406619

[B70] Luszczki J. J. Wojda E. Raszewski G. Głowniak K. Czuczwar S. J. (2008). Influence of Imperatorin on the Anticonvulsant Activity and Acute Adverse-Effect Profile of Lamotrigine in Maximal Electroshock-Induced Seizures and Chimney Test in Mice. Pharmacol. Rep. 60 (4), 566–573. 18799827

[B71] Marumoto S. Miyazawa M. (2010). Beta-Secretase Inhibitory Effects of Furanocoumarins from the Root of Angelica Dahurica. Phytother. Res. 24 (4), 510–513. 10.1002/ptr.2967 20041416

[B72] Matsuo Y. Yamaguchi E. Hakamata R. Ootomo K. Takatori K. Fukaya H. (2020). Benzofuran and Coumarin Derivatives from the Root of Angelica Dahurica and Their PPAR-γ Ligand-Binding Activity. Phytochemistry 173, 112301. 10.1016/j.phytochem.2020.112301 32092557

[B73] Mi C. Ma J. Wang K. S. Zuo H. X. Wang Z. Li M. Y. (2017). Imperatorin Suppresses Proliferation and Angiogenesis of Human Colon Cancer Cell by Targeting HIF-1α via the mTOR/p70S6K/4E-BP1 and MAPK Pathways. J. Ethnopharmacol. 203, 27–38. 10.1016/j.jep.2017.03.033 28341244

[B74] Najmanová I. Doseděl M. Hrdina R. Anzenbacher P. Filipský T. Říha M. (2015). Cardiovascular Effects of Coumarins besides Their Antioxidant Activity. Curr. Top. Med. Chem. 15 (9), 830–849. 10.2174/1568026615666150220112437 25697565

[B75] Nie H. Meng L. Z. Zhou J. Y. Fan X. F. Luo-Y. Zhang G. W. (2009). Imperatorin Is Responsible for the Vasodilatation Activity of Angelica Dahurica Var. Formosana Regulated by Nitric Oxide in an Endothelium-dependent Manner. Chin. J. Integr. Med. 15 (6), 442–447. 10.1007/s11655-009-0442-z 20082250

[B76] Oh H. Lee H. S. Kim T. Chai K. Y. Chung H. T. Kwon T. O. (2002). Furocoumarins from Angelica Dahurica with Hepatoprotective Activity on Tacrine-Induced Cytotoxicity in Hep G2 Cells. Planta. Med. 68 (5), 463–464. 10.1055/s-2002-32075 12058329

[B77] Pervin M. Hasnat M. A. Debnath T. Park S. R. Kim D. H. Lim B. O. (2014). Antioxidant, Anti-inflammatory and Antiproliferative Activity ofAngelica DahuricaRoot Extracts. J. Food Biochem. 38 (3), 281–292. 10.1111/jfbc.12046

[B78] Pfeifer I. Murauer A. Ganzera M. (2016). Determination of Coumarins in the Roots of Angelica Dahurica by Supercritical Fluid Chromatography. J. Pharm. Biomed. Anal. 129, 246–251. 10.1016/j.jpba.2016.07.014 27442886

[B79] Piao X. L. Park I. H. Baek S. H. Kim H. Y. Park M. K. Park J. H. (2004). Antioxidative Activity of Furanocoumarins Isolated from Angelicae Dahuricae. J. Ethnopharmacol. 93 (2-3), 243–246. 10.1016/j.jep.2004.03.054 15234759

[B80] Piao X. L. Yoo H. H. Kim H. Y. Kang T. L. Hwang G. S. Park J. H. (2006). Estrogenic Activity of Furanocoumarins Isolated from Angelica Dahurica. Arch. Pharm. Res. 29 (9), 741–745. 10.1007/bf02974073 17024846

[B81] Qi B. Yang W. Ding N. Luo Y. Jia F. Liu X. (2019). Pyrrole 2-carbaldehyde Derived Alkaloids from the Roots of Angelica Dahurica. J. Nat. Med. 73 (4), 769–776. 10.1007/s11418-019-01328-1 31209724

[B82] Qiao S. Y. Yao X. S. Wang Z. Y. (1996). Coumarins of the Roots of Angelica Dahurica. Planta. Med. 62 (6), 584. 10.1055/s-2006-957985 17252511

[B83] Sancho R. Márquez N. Gómez-Gonzalo M. Calzado M. A. Bettoni G. Coiras M. T. (2004). Imperatorin Inhibits HIV-1 Replication through an Sp1-dependent Pathway. J. Biol. Chem. 279 (36), 37349–37359. 10.1074/jbc.M401993200 15218031

[B84] Schinella G. R. Tournier H. A. Prieto J. M. Ríos J. L. Buschiazzo H. Zaidenberg A. (2002). Inhibition of Trypanosoma Cruzi Growth by Medical Plant Extracts. Fitoterapia 73 (7-8), 569–575. 10.1016/s0367-326x(02)00246-0 12490214

[B85] Seo W. D. Kim J. Y. Ryu H. W. Kim J. H. Han S.-I. Ra J.-E. (2013). Identification and Characterisation of Coumarins from the Roots of Angelica Dahurica and Their Inhibitory Effects against Cholinesterase. J. Funct. Foods 5 (3), 1421–1431. 10.1016/j.jff.2013.05.011

[B87] Shu P. Li J. Fei Y. Zhu H. Yu M. Liu A. (2020a). Isolation, Structure Elucidation, Tyrosinase Inhibitory, and Antioxidant Evaluation of the Constituents from Angelica Dahurica Roots. J. Nat. Med. 74 (2), 456–462. 10.1007/s11418-019-01375-8 31773388

[B88] Shu P. Li J. Fei Y. Zhu H. Zhang L. Niu H. (2020b). Angelicosides I-IV, Four Undescribed Furanocoumarin Glycosides from Angelica Dahurica Roots and Their Tyrosinase Inhibitory Activities. Phytochem. Lett. 36, 32–36. 10.1016/j.phytol.2020.01.006

[B89] Sigurdsson S. Gudbjarnason S. (2013). Effect of Oral Imperatorin on Memory in Mice. Biochem. Biophys. Res. Commun. 441 (2), 318–320. 10.1016/j.bbrc.2013.10.036 24140410

[B90] Sumorek-Wiadro J. Zając A. Maciejczyk A. Jakubowicz-Gil J. (2020). Furanocoumarins in Anticancer Therapy - for and against. Fitoterapia 142, 104492. 10.1016/j.fitote.2020.104492 32032635

[B91] Sun J. Li H. Sun J. Liu H. Chen J. Wang C. (2017). Chemical Composition and Antimigraine Activity of Essential Oil of Angelicae Dahuricae Radix. J. Med. Food. 20 (8), 797–803. 10.1089/jmf.2016.3898 28731365

[B92] Tabanca N. Gao Z. Demirci B. Techen N. Wedge D. E. Ali A. (2014). Molecular and Phytochemical Investigation of Angelica Dahurica and Angelica Pubescentis Essential Oils and Their Biological Activity against *Aedes aegypti*, Stephanitis Pyrioides, and Colletotrichum Species. J. Agric. Food Chem. 62 (35), 8848–8857. 10.1021/jf5024752 25133520

[B93] Thanh P. N. Jin W. Song G. Bae K. Kang S. S. (2004). Cytotoxic Coumarins from the Root of Angelica Dahurica. Arch. Pharm. Res. 27 (12), 1211–1215. 10.1007/bf02975883 15646793

[B94] Wang C. M. Li H. Cui X. Y. (2009a). Mechanism of Underlying Analgesic Effect of Coumarin of Angdicae Dahuricae. Chin. J. Gerontol. 29 (22), 2904–2906.

[B95] Wang D. Meng Y. Wang C. Wang X. Blasi F. (2020). Antioxidant Activity and Sensory Improvement of Angelica Dahurica Cv. Yubaizhi Essential Oil on Sunflower Oil during High-Temperature Storage. Processes 8 (4), 403. 10.3390/pr8040403

[B96] Wang G. H. Chen C. Y. Tsai T. H. Chen C. K. Cheng C. Y. Huang Y. H. (2017). Evaluation of Tyrosinase Inhibitory and Antioxidant Activities of Angelica Dahurica Root Extracts for Four Different Probiotic Bacteria Fermentations. J. Biosci. Bioeng. 123 (6), 679–684. 10.1016/j.jbiosc.2017.01.003 28254340

[B97] Wang H. L. Wang C. M. Cui X. Y. (2009). Studies on Analgesic Site and Mechanisms Underlying of Coumarins of Angdicae Dahuricae. Chin. J. Gerontol. 29 (15), 1902–1904.

[B98] Wang H. Wang X. Zhou L. Zhang S. An L. Bao J. (2021). Structural Characteristics and *In Vitro* and *In Vivo* Immunoregulatory Properties of a Gluco-Arabinan from Angelica Dahurica. Int. J. Biol. Macromol. 183, 90–100. 10.1016/j.ijbiomac.2021.04.077 33872613

[B99] Wang N. Wu L. Cao Y. Wang Y. Zhang Y. (2013). The Protective Activity of Imperatorin in Cultured Neural Cells Exposed to Hypoxia Re-oxygenation Injury via Anti-apoptosis. Fitoterapia 90, 38–43. 10.1016/j.fitote.2013.07.007 23856091

[B100] Wang N. H. Yoshizaki K. Baba K. (2001). Seven New Bifuranocoumarins, Dahuribirin A-G, from Japanese Bai Zhi. Chem. Pharm. Bull. (Tokyo) 49 (9), 1085–1088. 10.1248/cpb.49.1085 11558591

[B101] Wang T.-t. Jin H. Li Q. Cheng W.-m. Hu Q.-q. Chen X.-h. (2007). Isolation and Simultaneous Determination of Coumarin Compounds in Radix Angelica Dahurica. Chroma 65 (7-8), 477–481. 10.1365/s10337-007-0185-y

[B102] Wang Y. J. Yan H. Huang S. L. Wang G. Q. Yang M. Q. Peng G. P. (2020). Establishment of HPLC Fingerprint of Angelica Sinensis and Evaluation of Chemometrics. Chin. Tradit. Pat. Med. 42 (2), 514–519. doi:10.3969 /j.issn.1001-1528.2020.02.049

[B103] Wei W. Wu X. W. Deng G. G. Yang X. W. (2016). Anti-inflammatory Coumarins with Short- and Long-Chain Hydrophobic Groups from Roots of Angelica Dahurica Cv. Hangbaizhi. Phytochemistry 123, 58–68. 10.1016/j.phytochem.2016.01.006 26775737

[B104] Wu M. Li T. Chen L. Peng S. Liao W. Bai R. (2016). Essential Oils from Inula Japonica and Angelicae Dahuricae Enhance Sensitivity of MCF-7/ADR Breast Cancer Cells to Doxorubicin via Multiple Mechanisms. J. Ethnopharmacol. 180, 18–27. 10.1016/j.jep.2016.01.015 26795076

[B105] Xie Y. Chen Y. Lin M. Wen J. Fan G. Wu Y. (2007). High-performance Liquid Chromatographic Method for the Determination and Pharmacokinetic Study of Oxypeucedanin Hydrate and Byak-Angelicin after Oral Administration of Angelica Dahurica Extracts in Mongrel Dog Plasma. J. Pharm. Biomed. Anal. 44 (1), 166–172. 10.1016/j.jpba.2007.02.002 17344014

[B106] Xie Y. Zhao W. Zhou T. Fan G. Wu Y. (2010). An Efficient Strategy Based on MAE, HPLC-DAD-ESI-MS/MS and 2D-Prep-HPLC-DAD for the Rapid Extraction, Separation, Identification and Purification of Five Active Coumarin Components from Radix Angelicae Dahuricae. Phytochem. Anal. 21 (5), 473–482. 10.1002/pca.1222 20931624

[B107] Xu S. F. Ye Y. P. Li X. Y. Chen F. Y. (2011). Chemical Composition and Antioxidant Activities of Different Polysaccharides from the Roots of Angelica Dahurica. Chem. Biodivers. 8 (6), 1121–1131. 10.1002/cbdv.201000233 21674784

[B108] Yang L. Li Q. Feng Y. Qiu D. (2020). Simultaneous Determination of Three Coumarins in Angelica Dahurica by 1H-qNMR Method: A Fast and Validated Method for Crude Drug Quality Control. J. Anal. Methods. Chem. 2020, 8987560. 10.1155/2020/8987560 32280555 PMC7128064

[B109] Yang W. Q. Song Y. L. Zhu Z. X. Su C. Zhang X. Wang J. (2015). Anti-inflammatory Dimeric Furanocoumarins from the Roots of Angelica Dahurica. Fitoterapia 105, 187–193. 10.1016/j.fitote.2015.07.006 26183116

[B110] Yang W. Q. Zhu Z. X. Song Y. L. Qi B. W. Wang J. Su C. (2017). Dimeric Furanocoumarins from the Roots of Angelica Dahurica. Nat. Prod. Res. 31 (8), 870–877. 10.1080/14786419.2016.1250090 27784175

[B111] Zhang F. Q. Jiang J. L. Zhang J. T. Niu H. Fu X. Q. Zeng L. L. (2020). Current Status and Future Prospects of Stem Cell Therapy in Alzheimer's Disease. Neural. Regen. Res. 15 (2), 242–250. 10.4103/1673-5374.265544 31552889 PMC6905342

[B112] Zhang H. Gong C. Lv L. Xu Y. Zhao L. Zhu Z. (2009). Rapid Separation and Identification of Furocoumarins in Angelica Dahurica by High-Performance Liquid Chromatography with Diode-Array Detection, Time-Of-Flight Mass Spectrometry and Quadrupole Ion Trap Mass Spectrometry. Rapid Commun. Mass Spectrom. 23 (14), 2167–2175. 10.1002/rcm.4123 19530154

[B113] Zhang J. J. Yin Z. H. Hu M. Y. Wang J. M. Kang W. Y. (2018). Bioactivity-guided Isolation of α-glucosidase Inhibitory and Tyrosinase Active Constituents in Angelica Dahurica Roots. Curr. Top. Nutraceut. R. 16 (2), 165–171.

[B114] Zhang L. Wei W. Yang X.-W. (2017a). Simultaneous Quantification of Nine New Furanocoumarins in Angelicae Dahuricae Radix Using Ultra-fast Liquid Chromatography with Tandem Mass Spectrometry. Molecules 22 (2), 322. 10.3390/molecules22020322 PMC6155589 28230757

[B115] Zhang X. Feng J. Mu K. Ma H. Niu X. Liu C. (2005). Effects of Single Herbal Drugs on Adhesion and Migration of Melanocytes. J. Tradit. Chin. Med. 25 (3), 219–221. 16334729

[B116] Zhang X. Li W. Abudureheman A. Cheng T. Peng P. (2017b). Imperatorin Possesses Notable Anti-inflammatory A-ctivity I-n vitro and I-n vivo through I-nhibition of the NF-κB P-athway. Mol. Med. Rep. 16 (6), 8619–8626. 10.3892/mmr.2017.7706 28990061 PMC5779915

[B117] Zhang Y. Cao Y. Zhan Y. Duan H. He L. (2010). Furanocoumarins-imperatorin Inhibits Myocardial Hypertrophy Both *In Vitro* and *In Vivo* . Fitoterapia 81 (8), 1188–1195. 10.1016/j.fitote.2010.07.023 20691250

[B118] Zhang Y. B. Deng G. G. Wang T. X. Liu L. Yang X. W. (2019). Tissue Distribution Study of Angelica Dahurica Cv. Yubaizhi in Rat by Ultra-performance Liquid Chromatography with Tandem Mass Spectrometry. J. Pharm. Biomed. Anal. 174, 43–49. 10.1016/j.jpba.2019.05.046 31153136

[B119] Zhao A.-h. Yang X.-w. (2018). New Coumarin Glucopyranosides from Roots of Angelica Dahurica. Chin. Herb. Med. 10 (1), 103–106. 10.1016/j.chmed.2018.01.008

[B120] Zhao G. Peng C. Du W. Wang S. (2013). Pharmacokinetic Study of Eight Coumarins of Radix Angelicae Dahuricae in Rats by Gas Chromatography-Mass Spectrometry. Fitoterapia 89, 250–256. 10.1016/j.fitote.2013.06.007 23774663

[B121] Zhao X. z. Feng X. Jia X. Wang M. Shan Y. Dong Y. (2007b). New Coumarin Glucoside from Angelica Dahurica. Chem. Nat. Compd. 43 (4), 399–401. 10.1007/s10600-007-0148-4

[B122] Zhao X. Z. Feng X. Jia X. D. Dong Y. F. Wang M. (2007a). Neolignan Glycoside from Angelica Dahurica. Chin. Chem. Lett. 18 (2), 168–170. 10.1016/j.cclet.2006.12.011

[B123] Zheng G. Y. Ma Y. Y. Lu X. L. Zhai M. Guo P. L. (2012). Effect of Sulfuring on the Acute Toxicity of the Angelica Extract. Pharm. Clin. Chin. Mat. Med. 3 (2), 43–44. 50.

[B124] Zheng X. Zhang X. Sheng X. Yuan Z. Yang W. Wang Q. (2010). Simultaneous Characterization and Quantitation of 11 Coumarins in Radix Angelicae Dahuricae by High Performance Liquid Chromatography with Electrospray Tandem Mass Spectrometry. J. Pharm. Biomed. Anal. 51 (3), 599–605. 10.1016/j.jpba.2009.09.030 19879083

[B125] Zheng Y. M. Lu A. X. Shen J. Z. Kwok A. H. Ho W. S. (2016a). Imperatorin Exhibits Anticancer Activities in Human Colon Cancer Cells via the Caspase Cascade. Oncol. Rep. 35 (4), 1995–2002. 10.3892/or.2016.4586 26794238

[B126] Zheng Y. M. Shen J. Z. Wang Y. Lu A. X. Ho W. S. (2016b). Anti-oxidant and Anti-cancer Activities of Angelica Dahurica Extract via Induction of Apoptosis in Colon Cancer Cells. Phytomedicine 23 (11), 1267–1274. 10.1016/j.phymed.2015.11.008 26776960

[B127] Zhu H. Sun Y. Xia Z. N. Gao T. T. Du C. H. Lu W. (2013). Pharmacology Studies on Acute Toxicity and Safety of Imperatorin. J. Pharm. Pract. 31 (6), 428–431. 475. 10.3969/j.issn.1006-0111.2013.06.008

