# Supplementary material for: The Angelica dahurica: A Review of Traditional Uses, Phytochemistry and Pharmacology
Source: Front Pharmacol. 2022 Jul 1;13:896637. doi: 10.3389/fphar.2022.896637 (PMC9283917; doi:10.3389/fphar.2022.896637)
Supplement: Supplementary file 1 [file Table1.docx]

**SUPPLEMENTARY TABLE 1**

Coumarins isolated from *A. dahurica*.

|  | |  |  |  | | |  | |  |
| --- | --- | --- | --- | --- | --- | --- | --- | --- | --- |
| Scopletin (3) | | Isoimperatorin (19) | Oxypeucedanin (20) | Imperatorin (22) | | | Phellopterin (26) | | Byakangelicin (41) |
| No | Names | | | | Plant pats | Formulas | | Refs | |
| **Simple coumarins** | | | | | | | | | |
| 1 | Coumarin | | | | Roots | C_9_H_6_O_2_ | | (Zhao et al., 2013) | |
| 2 | Umbelliferone | | | | Roots | C_9_H_6_O_3_ | | (Chen et al., 2021) | |
| 3 | Scopoletin | | | | Roots, Stems | C_10_H_8_O_4_ | | (Chen et al., 2021; Kwon et al., 2002) | |
| 4 | 6,7-Dimethoxycoumarin | | | | Roots | C_11_H_10_O_4_ | | (Chen et al., 2021) | |
| 5 | 5,7-Dimethoxycoumarin | | | | Roots | C_11_H_10_O_4_ | | (Chen et al., 2021) | |
| 6 | Fraxidin | | | | Roots | C_11_H_10_O_5_ | | (Chen et al., 2021) | |
| 7 | 5,6,7-Trimethoxy-2H-benzopyran-2-one | | | | Roots | C_12_ H_12_ O_5_ | | (Chen et al., 2021) | |
| 8 | 6,7,8-Trimethoxycoumarin | | | | Roots | C_12_H_12_O_5_ | | (Chen et al., 2021) | |
| 9 | 7-Hydroxy-6-(2′,3′,4′-trihydroxy-isopentanyl)-coumarin | | | | Roots | C_14_H_16_O_6_ | | (Matsuo et al., 2020) | |
| 10 | 2′-Deoxymeranzin hydrate | | | | Roots | C_15_H_18_O_4_ | | (Zhang et al., 2018) | |
| 11 | Osthenol | | | | Roots | C_14_H_14_O_3_ | | (Xie et al., 2010) | |
| 12 | Auraptenol | | | | Roots | C_15_H_16_O_4_ | | (Xie et al., 2010) | |
| 13 | Suberosin | | | | Fruits | C_15_H_16_O_3_ | | (Zhang et al., 2009) | |
| 14 | Angelol H | | | | Stems | C_20_H_24_O_7_ | | (Kwon et al., 2002) | |
| 15 | Angelol I | | | | Stems | C_20_H_26_O_7_ | | (Kwon et al., 2002) | |
| 16 | 6-[1(S), 2(R)-2, 3-dihydroxy-1-methoxy-3-methylbutyl]-7-methoxycoumarin | | | | Stems | C_16_H_20_O_6_ | | (Kwon et al., 2002) | |
| 17 | Osthol | | | | Roots | C_15_H_16_O_3_ | | (Thanh et al., 2004) | |
| 18 | 7-Demethylsuberosin | | | | Roots | C_14_H_14_O_3_ | | (Fujiwara et al., 1980) | |
| **Furanocoumarins** | | | | | | | | | |
| 19 | Isoimperatorin | | | | Roots | C_16_H_14_O_4_ | | (Lee, B.W. et al., 2020) | |
| 20 | Oxypeucedanin | | | | Roots | C_16_H_14_O_5_ | | (Lee, B.W. et al., 2020) | |
| 21 | Oxypeucedanin hydrate | | | | Roots | C_16_H_16_O_6_ | | (Lee, B.W. et al., 2020) | |
| 22 | Imperatorin | | | | Roots | C_16_H_14_O_4_ | | (Lee, B.W. et al., 2020) | |
| 23 | Psoralen | | | | Roots | C_11_H_6_O_3_ | | (Lee, S.H. et al., 2020) | |
| 24 | Xanthotoxin | | | | Roots | C_12_H_8_O_4_ | | (Lee, S.H. et al., 2020) | |
| 25 | Bergapten | | | | Roots | C_12_H_8_O_4_ | | (Lee, S.H. et al., 2020) | |
| 26 | Phellopterin | | | | Roots | C_17_H_16_O_5_ | | (Lee, S.H. et al., 2020) | |
| 27 | Cnidilin | | | | Roots | C_17_H_16_O_5_ | | (Lee, S.H. et al., 2020) | |
| 28 | Marmesin | | | | Roots | C_14_H_14_O_4_ | | (Shu et al., 2020a) | |
| 29 | Xanthoarnol | | | | Roots | C_14_H_14_O_5_ | | (Shu et al., 2020a) | |
| 30 | Pangelin | | | | Roots | C_16_H_14_O_5_ | | (Shu et al., 2020a) | |
| 31 | Xanthotoxol | | | | Roots | C_11_H_6_O_4_ | | (Shu et al., 2020a) | |
| 32 | Isopimpinellin | | | | Roots | C_13_H_10_O_5_ | | (Shu et al., 2020a) | |
| 33 | Heraclenin | | | | Roots | C_16_H_14_O_5_ | | (Shu et al., 2020a) | |
| 34 | Byakangelicol | | | | Roots | C_17_H_16_O_6_ | | (Shu et al., 2020a) | |
| 35 | Dahuribirin H | | | | Roots | C_33_H_30_O_10_ | | (Kang et al., 2019) | |
| 36 | Dahuribirin I | | | | Roots | C_33_H_30_O_10_ | | (Kang et al., 2019) | |
| 37 | (2′S)-(+)-5-(2′-Hydroxy-3′-methylbut-3′-enyloxy)-8-(3′′-methylbut-2′′-enyloxy)-psoralen | | | | Roots | C_21_H_22_O_6_ | | (Kang et al., 2019) | |
| 38 | (2′R)-(+)-5-(2′,3′-Epoxy-3′-methylbutoxy)-8-(3′′-methylbut-2′′-enyloxy)-psoralen | | | | Roots | C_21_H_22_O_6_ | | (Kang et al., 2019) | |
| 39 | 5-Methoxy-8-((Z)-4′-(3′′-methylbutanoate)-3′-methylbut-2′-enyloxy)-psoralen | | | | Roots | C_22_H_24_O_7_ | | (Kang et al., 2019) | |
| 40 | 5-Methoxy-8-hydroxy psoralen | | | | Roots | C_12_H_8_O_5_ | | (Kang et al., 2019) | |
| 41 | Byakangelicin | | | | Roots | C_17_H_18_O_7_ | | (Kang et al., 2019) | |
| 42 | t-OMe-Byakangelicin | | | | Roots | C_18_H_21_O_7_ | | (Kang et al., 2019) | |
| 43 | 5-(2′,3′-Dihydroxy-3′-methylbutyloxy)-8-(3″-methylbut-2″-enyloxy)-psoralen | | | | Roots | C_21_H_24_O_7_ | | (Kang et al., 2019) | |
| 44 | 5-(3-Methylbut-2-enyloxy)-8-(2,3-dihydroxy-3-methylbutoxy)-psoralen | | | | Roots | C_21_H_24_O_7_ | | (Kang et al., 2019) | |
| 45 | Pabulenol | | | | Roots | C_16_H_14_O_5_ | | (Zhang et al., 2018) | |
| 46 | Isogosferol | | | | Roots | C_16_H_14_O_5_ | | (Zhang et al., 2018) | |
| 47 | Heraclenol | | | | Roots | C_16_H_16_O_6_ | | (Zhang et al., 2018) | |
| 48 | Oxypeucedanin hydrate acetonide | | | | Roots | C_19_H_20_O_6_ | | (Thanh et al., 2004) | |
| 49 | Sec-O-acetylbyakangelicin | | | | Roots | C_19_H_20_O_8_ | | (Kimura and Okuda, 1997) | |
| 50 | Isobyakangelicin | | | | Fruits | C_17_H_18_O_7_ | | (Zhang et al., 2009) | |
| 51 | Senbyakangelicol | | | | Roots | C_21_H_23_O_7_ | | (Seo et al., 2013) | |
| 52 | Andafocoumarins A | | | | Roots | C_28_H_40_O_6_ | | (Zhang, L. et al., 2017) | |
| 53 | Andafocoumarin B | | | | Roots | C_28_H_40_O_6_ | | (Zhang, L. et al., 2017) | |
| 54 | Andafocoumarin C | | | | Roots | C_29_H_42_O_7_ | | (Zhang, L. et al., 2017) | |
| 55 | Andafocoumarins D | | | | Roots | C_30_H_44_O_6_ | | (Zhang, L. et al., 2017) | |
| 56 | Andafocoumarins E | | | | Roots | C_30_H_44_O_6_ | | (Zhang, L. et al., 2017) | |
| 57 | Andafocoumarins F | | | | Roots | C_31_H_46_O_7_ | | (Zhang, L. et al., 2017) | |
| 58 | Andafocoumarins G | | | | Roots | C_34_H_50_O_6_ | | (Zhang, L. et al., 2017) | |
| 59 | Andafocoumarins H | | | | Roots | C_34_H_50_O_6_ | | (Zhang, L. et al., 2017) | |
| 60 | Andafocoumarins J | | | | Roots | C_35_H_52_O_7_ | | (Zhang, L. et al., 2017) | |
| 61 | Andafocoumarins I | | | | Roots | C_35_H_52_O_7_ | | (Wei et al., 2016) | |
| 62 | 5-(3''-Hydroxy-3''-methylbutyl)-8-hydroxyfuranocoumarin | | | | Roots | C_16_H_16_O_5_ | | (Wei et al., 2016) | |
| 63 | Isobyakangelicin hydrate-3″-ethyl ether | | | | Roots | C_19_H_22_O_7_ | | (Wei et al., 2016) | |
| 64 | Oxypeucedanin hydrate-3″-butyl ether | | | | Roots | C_20_H_22_O_7_ | | (Choi et al., 2005) | |
| 65 | Pimpinellin | | | | Roots | C_13_H_10_O_5_ | | (Li and Wu, 2017) | |
| 66 | Angelicin | | | | Roots | C_11_H_6_O_3_ | | (Pfeifer et al., 2016) | |
| 67 | 9-Hydroxy-4-methoxypsoralen | | | | Roots | C_12_H_8_O_5_ | | (Piao et al., 2006) | |
| 68 | Dahuribiethrins H | | | | Roots | C_33_H_28_O_10_ | | (Yang et al., 2017) | |
| 69 | Dahuribiethrins I | | | | Roots | C_33_H_28_O_10_ | | (Yang et al., 2017) | |
| 70 | Dahuribiethrins J | | | | Roots | C_33_H_28_O_10_ | | (Yang et al., 2017) | |
| 71 | Dahurinol A | | | | Roots | C_31_H_50_O_5_ | | (Yang et al., 2017) | |
| 72 | Angdahuricaol A | | | | Roots | C_33_H_32_O_12_ | | (Bai et al., 2016) | |
| 73 | Angdahuricaol B | | | | Roots | C_33_H_32_O_12_ | | (Bai et al., 2016) | |
| 74 | Angdahuricaol C | | | | Roots | C_33_H_32_O_12_ | | (Bai et al., 2016) | |
| 75 | 5,8-Bis-(2,3-dihydroxy-3-methylbutyloxy)-psoralen | | | | Roots | C_21_H_26_O_9_ | | (Matsuo et al., 2020) | |
| 76 | Pabularinone | | | | Roots | C_16_H_14_O_5_ | | (Bai et al., 2016) | |
| 77 | Neobyakangelicol | | | | Roots | C_17_H_16_O_6_ | | (Bai et al., 2016) | |
| 78 | Aviprin | | | | Roots | C_16_H_16_O_6_ | | (Bai et al., 2016) | |
| 79 | Bergaptol | | | | Roots | C_11_H_6_O_4_ | | (Bai et al., 2016) | |
| 80 | 5-Methoxy-8-(2-hydroxy-3-buthoxy-3-methylbutyloxy)-psoralen | | | | Roots | C_21_H_26_O_7_ | | (Hua et al., 2008) | |
| 81 | Knidilin | | | | Roots | C_17_H_16_O_5_ | | (Baek et al., 2000) | |
| 82 | Columbianetin | | | | Roots | C_14_H_14_O_4_ | | (Zhang et al., 2019) | |
| 83 | Dahuribiethrins A | | | | Roots | C_33_H_31_O_11_ | | (Yang et al., 2015) | |
| 84 | Dahuribiethrins B | | | | Roots | C_33_H_31_O_11_ | | (Yang et al., 2015) | |
| 85 | Dahuribiethrins C | | | | Roots | C_32_H_29_O_10_ | | (Yang et al., 2015) | |
| 86 | Dahuribiethrins D | | | | Roots | C_33_H_31_O_11_ | | (Yang et al., 2015) | |
| 87 | Dahuribiethrins E | | | | Roots | C_33_H_31_O_11_ | | (Yang et al., 2015) | |
| 88 | Dahuribiethrins F | | | | Roots | C_33_H_31_O_11_ | | (Yang et al., 2015) | |
| 89 | Dahuribiethrins G | | | | Roots | C_33_H_31_O_11_ | | (Yang et al., 2015) | |
| 90 | Dahuribiscoumarin | | | | Roots | C_27_H_17_O_9_ | | (Deng et al., 2015b) | |
| 91 | Isodemethylfuropinarine | | | | Roots | C_16_H_14_O_4_ | | (Deng et al., 2015b) | |
| 92 | Demethylfuropinarine | | | | Roots | C_16_H_14_O_4_ | | (Deng et al., 2015b) | |
| 93 | 8-Geranyloxypsoralen | | | | Roots | C_21_H_22_O_4_ | | (Xie et al., 2010) | |
| 94 | 8-Geranoxy-5-methoxypsoralen | | | | Roots | C_22_H_24_O_5_ | | (Xie et al., 2010) | |
| 95 | Oxyalloimperatorin | | | | Roots | C_17_H_16_O_5_ | | (Bai et al., 2016) | |
| 96 | Alloimperatorin | | | | Roots | C_16_H_14_O_4_ | | (Bai et al., 2016) | |
| 97 | Isooxypeucedanin | | | | Roots | C_16_H_14_O_5_ | | (Bai et al., 2016) | |
| 98 | Apaensin | | | | Roots | C_17_H_16_O_6_ | | (Bai et al., 2016) | |
| 99 | Isobayakangelicol | | | | Roots | C_17_H_16_O_6_ | | (Li et al., 2014) | |
| 100 | t-OMe-oxypeucedanin hydrate | | | | Roots | C_17_H_18_O_6_ | | (Seo et al., 2013) | |
| 101 | Bergamottin | | | | Roots | C_21_H_22_O_4_ | | (Li et al., 2014) | |
| 102 | 8-Geranoxypsoralen | | | | Roots | C_21_H_22_O_4_ | | (Li et al., 2014) | |
| 103 | Cnidicin | | | | Roots | C_21_H_22_O_5_ | | (Li et al., 2014) | |
| 104 | Anhydrobyakangelicin | | | | Roots | C_17_H_16_O_6_ | | (Li et al., 2014) | |
| 105 | 5-Hydroxy xanthotoxin | | | | Roots | C_12_H_8_O_5_ | | (Li et al., 2014) | |
| 106 | Alloisoimperatorin | | | | Roots | C_16_H_14_O_4_ | | (Li et al., 2014) | |
| 107 | Bergaptol | | | | Roots | C_11_H_6_O_4_ | | (Li et al., 2014) | |
| 108 | Isogospherol | | | | Roots | C_16_H_14_O_5_ | | (Li et al., 2014) | |
| 109 | Iso-tert-O-methylbyakangelicin | | | | Fruits | C_18_H_20_O_7_ | | (Zhang et al., 2009) | |
| 110 | 5-Demethoxy-isodahuribirin A | | | | Fruits | C_32_H_28_O_9_ | | (Zhang et al., 2009) | |
| 111 | Isodahuribirin A | | | | Fruits | C_33_H_30_O_10_ | | (Zhang et al., 2009) | |
| **Coumarin glucosides** | | | | | | | | | |
| 112 | Angelicoside I | | | | Roots | C_23_H_28_O_11_ | | (Shu et al., 2020b) | |
| 113 | Angelicoside Ⅱ | | | | Roots | C_23_H_28_O_11_ | | (Shu et al., 2020b) | |
| 114 | Angelicoside Ⅲ | | | | Roots | C_29_H_38_O_15_ | | (Shu et al., 2020b) | |
| 115 | Angelicoside IV | | | | Roots | C_29_H_38_O_17_ | | (Shu et al., 2020b) | |
| 116 | Tert-O-β-D-apiofuranosyl-(1→6)-O-β-D-glucopyranosyl-byakangelicin | | | | Roots | C_27_H_34_O_15_ | | (Shu et al., 2020b) | |
| 117 | 1′-O-β-D-Glucopyranosyl-(2R,3S)-3-hydroxynodakenetin | | | | Roots | C_20_H_24_O_10_ | | (Shu et al., 2020b) | |
| 118 | Decuroside IV | | | | Roots | C_25_H_32_O_13_ | | (Shu et al., 2020b) | |
| 119 | Nodakenin | | | | Roots | C_20_H_24_O_9_ | | (Shu et al., 2020b) | |
| 120 | Xanthotoxol 8-O-β-D-glucopyranoside | | | | Roots | C_17_H_16_O_9_ | | (Shu et al., 2020b) | |
| 121 | Scopolin | | | | Roots | C_16_H_18_O_9_ | | (Deng et al., 2015a) | |
| 122 | Angelicoside A | | | | Roots | C_20_H_24_O_8_ | | (Shu et al., 2020a) | |
| 123 | (-)-Marmesinin | | | | Roots | C_20_H_24_O_9_ | | (Zhao and Yang, 2018) | |
| 124 | β-D-Glucosyl-6′-(β-D-apiosyl) columbianetin | | | | Roots | C_25_H_32_O_13_ | | (Shu et al., 2020b) | |
| 125 | 3′-Hydroxymarmesinin | | | | Roots | C_20_H_24_O_10_ | | (Choi et al., 2005) | |
| 126 | Isopraeroside IV | | | | Roots | C_20_H_24_O_9_ | | (Choi et al., 2005) | |
| 127 | 2′-O-β-D-Apiofuranosyl-(1→6)-β-D-gluco-pyranosylpeucedanol | | | | Roots | C_25_H_34_O_14_ | | (Jia et al., 2008b) | |
| 128 | Dahurin B | | | | Roots | C_22_H_26_O_11_ | | (Zhao et al., 2007b) | |
| 129 | Tert-O-β-D-apiofuranosyl-(1→6)-O-β-D-glucopyranosyl-oxypeucedanin hydrate | | | | Roots | C_27_H_34_O_15_ | | (Jia et al., 2008a) | |
| 130 | Sec-O-β-D-apiofuranosyl-(1→6)-O-β-D-glucopyranosyl-oxypeucedanin hydrate | | | | Roots | C_27_H_34_O_15_ | | (Jia et al., 2008a) | |
| 131 | Xanthoarnol-3'-O-β-D-glucopyranoside | | | | Roots | C_20_H_24_O_10_ | | (Zhao and Yang, 2018) | |
| 132 | Angedahuricoside A | | | | Roots | C_21_H_28_O_11_ | | (Zhao and Yang, 2018) | |
| 133 | Angedahuricoside B | | | | Roots | C_21_H_28_O_11_ | | (Zhao and Yang, 2018) | |
| 134 | Isofraxidin-7-O-β-D-glucopyranoside | | | | Roots | C_17_H_20_O_10_ | | (Zhao and Yang, 2018) | |
| 135 | Fraxidin-8-O-β-D-glucopyranoside | | | | Roots | C_17_H_20_O_10_ | | (Zhao and Yang, 2018) | |
| 136 | (2'S, 3'R)-3'-Hydroxymarmesinin | | | | Roots | C_20_H_24_O_10_ | | (Zhao and Yang, 2018) | |
| 137 | Hyuganoside V | | | | Roots | C_20_H_28_O_10_ | | (Zhao and Yang, 2018) | |
| 138 | 1′-O-β-D-Glucopyranosyl-3′-hydroxynodakenetin | | | | Roots | C_20_H_24_O_10_ | | (Matsuo et al., 2020) | |
| 139 | (3′S)-Hydroxy-nodakenetin 4′-O-β-D-apiofuranosyl-(1→6)-β-D-glucopyranoside | | | | Roots | C_25_H_32_O_14_ | | (Matsuo et al., 2020) | |
| 140 | Columbianetin 2′-O-β-D-glucopyranoside | | | | Roots | C_20_H_23_O_9_ | | (Matsuo et al., 2020) | |
| 141 | Columbianetin 2′-O-β-Dapiofuranosyl-(1→6)-β-D-glucopyranoside | | | | Roots | C_25_H_31_O_13_ | | (Matsuo et al., 2020) | |
| 142 | R-(+)-Oxypeucedanin hydrate sucrose ether | | | | Roots | C_28_H_36_O_16_ | | (Matsuo et al., 2020) | |
| 143 | S-(-)-Oxypeucedanin hydrate sucrose ether | | | | Roots | C_28_H_36_O_16_ | | (Matsuo et al., 2020) | |
| 144 | Byakangelicin 3″-O-β-D-glucopyranoside | | | | Roots | C_23_H_30_O_12_ | | (Matsuo et al., 2020) | |
| 145 | Byakangelicin 3″-O-β-D-apiofuranosyl-(1 → 6)-β-D-glucopyranoside | | | | Roots | C_28_H_38_O_16_ | | (Matsuo et al., 2020) | |
| 146 | R-(+)-Byakangelicin sucrose ether | | | | Roots | C_29_H_38_O_17_ | | (Matsuo et al., 2020) | |
| 147 | 3″-O-β-D-Glucopyranosyl-5,8-bis(2,3-dihydroxy-3-methylbutyloxy)-psoralen | | | | Roots | C_27_H_36_O_14_ | | (Matsuo et al., 2020) | |
| **Other coumarins** | | | | | | | | | |
| 148 | Angedahurin A | | | | Roots | C_15_H_16_O_3_ | | (Chen et al., 2021) | |
| 149 | (-)-Hydroxydecursinol | | | | Roots | C_14_H_13_O_5_ | | (Deng et al., 2015b) | |
| 150 | (+)-Decursinol | | | | Roots | C_14_H_14_O_4_ | | (Deng et al., 2015b) | |
| **Coumarin derivatives** | | | | | | | | | |
| 151 | 2″-Sulfo-(±)-oxypeucedanin hydrate | | | | Roots | C_16_H_16_O_9_S | | (Matsuo et al., 2020) | |
| 152 | 3″-Sulfo-(±)-oxypeucedanin hydrate | | | | Roots | C_16_H_16_O_9_S | | (Matsuo et al., 2020) | |
| 153 | 2″-Sulfo-(±)-byakangelicin | | | | Roots | C_17_H_18_O_10_S | | (Matsuo et al., 2020) | |
