# Supplementary material for: The Angelica dahurica: A Review of Traditional Uses, Phytochemistry and Pharmacology
Source: Front Pharmacol. 2022 Jul 1;13:896637. doi: 10.3389/fphar.2022.896637 (PMC9283917; doi:10.3389/fphar.2022.896637)
Supplement: Supplementary file 2 [file Table2.docx]

**SUPPLEMENTARY TABLE 2**

The pharmacological activities of *A. dahurica* root*.*

| Pharmacological activities | Extracts/compounds | Doses/concentrations | Models | Effects | Targets or signaling pathways | Refs. |
| --- | --- | --- | --- | --- | --- | --- |
| Anti-inflammatory activity | 50% Ethanol extract | 1 and 100 mg/mL | Rat models of periodontitis | Decreasing pro-inflammatory factors | IL-1β, IL-6, IL-8, IFN-γ, NF-κB, COX-2 and iNOS | (Lee et al., 2017) |
|  | 70% Ethanol extract | 50 and 100 mg/kg b.w. | Ovalbumin-induced airway inflammation in asthmatic mice | Decreasing airway eosinophilia, cytokine levels, mucus and IgE | HO-1 | (Lee et al., 2011) |
|  | IMP | 15, 30 and 60 mg/kg b.w. | Dimethylbenzene‑induced ear edema in mice,  Acetic acid‑induced vascular permeability in mice,  Ball fralunoma weight cotton pellet‑induced granuloma in rats,  LPS-induced RAW 264.7 cells | Decreasing pro-inflammatory factors, inhibiting ear edema, vascular permeability and ball fralunoma weight | TNF‑α, IL‑6, IL‑1 iNOS, COX‑2, p65 and IκB | (Zhang, X.X. et al., 2017) |
|  | Compound 21, 25 26 and 41 | 20 μM | DNP-HAS-induced RBL‑2H3 cells | Decreasing histamine release, inhibiting inflammatory cytokines | TNF-α, IL-4, IL-1β and NF-κ B signaling | (Li and Wu, 2017) |
| Anti-tumor activity | 70% Ethanol extract | 100 and 200 μg/mL | Murine melanoma B16F10 cells | Inhibiting cell growth, migration, invasion and colony formation,  inducing cell apoptosis | MMP-2 and -9 | (Hwangbo et al., 2020) |
|  | Essential oils | 12.5 μg/mL | MCF-7/ADR breast cancer cells | Inhibiting the resistance of MCF-7/ADR cells to doxorubicin | ABCB1 | (Wu et al., 2016) |
|  | Ethyl acetate extract | 100-250 μg/mL | Colon cancer HT-29 cells | Inducing cell apoptosis | p53, p21, MDM2, Bcl, Bax and caspase 3/7 | (Zheng et al., 2016b) |
|  | IMP | 150 μM | Colon cancer HCT116 cells,  HCT116 xenograft mice | Inhibiting cell proliferation, angiogenesis and tumor growth | HIF-1α, mTOR/p70S6K/4E-BP1 and AMPK signaling pathways | (Mi et al., 2017) |
|  | IMP | 150 μM | Colon cancer HT‑29 cells | Inhibiting cell proliferation, Inducing cell apoptosis | p53 and caspase cascade | (Zheng et al., 2016a) |
|  | IMP | 0.1-1 μg/mL | Lung cancer H292 and A549 cells | Promoting cell anoikis | p53, Mcl-1, Bcl-2, and BAX | (Choochuay et al., 2013) |
|  | IMP | 25 and 50 mg/kg b.w. | Colorectal adenocarcinoma CT26 tumor-bearing mice | Mitigating cancer cachexia | STAT3 | (Chen et al., 2020) |
| Anti-oxidant activity | 70% Ethanol extract | N/A | DPPH solution | Scavenging DPPH radical | N/A | (Wang et al., 2017) |
|  | Compound 66 and 104 | N/A | DPPH solution | Scavenging DPPH radical | N/A | (Piao et al., 2004) |
|  | Compound 289, 290 and 291 | N/A | DPPH solution | Scavenging DPPH radical | N/A | (Shu et al., 2020a) |
|  | ADP1-ADP4 | N/A | N/A | Inhibiting MDA formation,  chelating Fe^2+^ and scavenging HO. radical | N/A | (Xu et al., 2011) |
| Analgesic activity | IMP | 2.45 mM | Formalin and capsaicin induced-rats | Alleviating pain | TRPV1 | (Chen et al., 2014) |
|  | Water extract | 100 mg/kg b.w. | Formalin, capsaicin and thermal induced-mice | Alleviating pain | TRPV1 | (Guo et al., 2019) |
|  | Coumarins | 30, 60 and 120 mg/kg b.w. | Formalin-induced mice | Alleviating pain | NO and β-EP | (Wang, C.M. et al., 2009; Wang, H.L. et al., 2009) |
| Anti-microbial activity | 70% Ethanol extract | N/A | *Trypanosoma cruzi* | Inhibiting the growth of *Trypanosoma cruzi* | N/A | (Schinella et al., 2002) |
|  | Water extract | 1 g/mL | *Mycoplasma hominis* | Inhibiting the growth of *Mycoplasma hominis* | N/A | (Che et al., 2005) |
|  | Falcarindiol | N/A | *Staphylococcal strains* | Inhibiting the growth of *Staphylococcal strains* | N/A | (Lechner et al., 2004) |
| Anti-viral activity | Compound 19, 20,21 and IMP | N/A | Influenza A viruses | Inhibiting influenza A viruses infection | N/A | (Lee, B.W. et al., 2020) |
|  | IMP | N/A | HIV-1 viruse | Inhibiting HIV-1 replication | Sp1 | (Sancho et al., 2004) |
| Effects on the cardiovascular system | 70% methanol extract | 1 mg/mL | Calcium-induced vasocontraction of rat aortic rings | Vasodilation | N/A | (Lee et al., 2015) |
|  | IMP | 15 and 30 mg/kg b.w. | HFFD-fed rats | Reducing blood pressure and heart rate values, alleviating changes in vascular morphology | Adiponectin receptor 1, eNOS and p47^phox^ | (Bunbupha et al., 2021) |
|  | IMP | N/A | Phenylephrine-induced mouse thoracic aorta | Vasodilatation | NO | (Nie et al., 2009) |
|  | IMP | 10, 30 and 100 μM | Potassium chloride (KCl) and endothelin-1-induced rat mesenteric arteries | Vasodilatation | 5-HT recptors | (He et al., 2007) |
|  | IMP | 25 and 6.25 mg/kg b.w. *in vivo*, 3, 10 and 30 μM *in vitro* | Spontaneous hypertensive rats,  ardiac muscle/CMC model | Attenuating pathological myocardial hypertrophy and cardiac fibrosis, inhibiting transition to heart failure, preventing cardiac myocyte protein synthesis and cell size | N/A | (Zhang et al., 2010) |
| Neuroprotective activity | IMP | 5 and 10 mg/kg b.w. | LPS-induced mice with poor memory retention | Ameliorating memory disturbances | BDNF | (Chowdhury et al., 2018) |
|  | IMP | 0.612 and 2.56μM *in vitro*, 5 and 10 mg/kg b.w. *in vivo* | Oxygen glucose deprivation/reperfusion-induced SH-SY5Y cells,  MCAO rats | Reducing infarct volume and increasing behavior ability | BDNF and p-ERK | (Wang et al., 2013) |
|  | IMP | 50 mg/kg b.w. | Electroshock-induced seizure mice | Enhancing the anticonvulsant activity of lamotrigine | N/A | (Luszczki et al., 2008) |
|  | IMP | 50 and 100 mg/kg b.w. | Electroshock-induced seizure mice | Increasing the MES threshold | N/A | (Luszczki et al., 2007) |
|  | Scopoletin | 2, 10 and 50 mg/kg b.w. | CFA-induced mice | Mitigating anxiety-like  behaviors | GABA_A_ receptors | (Luo et al., 2020) |
| Hepatoprotective activity | IMP and byakangelicin | N/A | Tacrine-induced Hep G2 cells | Inhibiting tacrine-induced cytotoxicity | N/A | (Oh et al., 2002) |
|  | IMP | 50 and 100 mg/kg b.w. | Acetaminophen overdose-induced mice with acute liver injury | Reducing mortality, ALT and AST in serum and centrilobular hepatic necrosis | SIRT1, FXR | (Gao et al., 2020) |
|  | Byakangelicin | 100 mg/kg b.w. | Carbon tetrachloride-induced mice with liver fibrosis and damage | Inhibiting the deposition of collagen and α-SMA, decreasing ALT and AST in serum | ASK-1/JNK signaling | (Li et al., 2020) |
| Effects on skin diseases | AD | 1.8 g/kg b.w. | db/db mice | Promoting would healing and angiogenesis | PI3K/AKT and HIF-1α/PDGF-β signalings | (Guo et al., 2020) |
|  | 70% Ethanol extract | 2.5 mg/mL | Melanocytes | Improving the adhesion of melanocytes to fibronectin, stimulating the migration of melanocytes | N/A | (Zhang et al., 2005) |
|  | Methanol extract and IMP | 10, 20 and 40 μM for IMP, 0.4, 2, 10 and 50 μg/mL for extract | IGF-1-indued sebocytes | Inhibiting sebum production | Akt, PPAR-γ and SREBP-1 | (Hwang et al., 2016) |
|  | IMP and isoimperatorin | N/A | B16 melanoma cells | Preventing tyrosinase synthesis | N/A | (Cho et al., 2006) |
| Regulation of lipid metabolism | 70% Ethanol extract | 800 mg/kg b.w. *in vivo* 400 μg/mL *in vitro* | HFFD–induced hyperlipidemia mice,  50% FBS-fed HepG2 cells | Reducing TC and TG, promoting the activity of total hepatic lipolysis | PPARγ and LIPC | (Lu et al., 2016) |
| Anti-diabetic activity | Phellopterin, ethyl acetate extract | 0.5, 1 and 2 mg/kg b.w. *in vivo*, 50 μg/mL *in vitro* | HFD/STZ-induced type Ⅱ diabetic mice, 3T3-L1 preadipocytes | Decreasing blood glucose, TC and TG, inducing adipocytes differentiation | PPARγ | (Han et al., 2018) |
| Immunoregulatory activity | ADP80-2 | 25, 50 and 100 μg/mL | Mouse macrophage RAW264.7, zebrafish embryos | Promoting the phagocytosis of macrophage cells, the release of NO and the generation of cytokines, inducing the production of ROS and NO | N/A | (Wang et al., 2021) |
|  | ADP | 10, 30 and 100 μg/mL | Dendritic cells | Activating the functions of Dendritic cells | TLR4 , MAPKs and NF-κB | (Kim et al., 2013) |
