# Supplementary material for: The Angelica dahurica: A Review of Traditional Uses, Phytochemistry and Pharmacology
Source: Front Pharmacol. 2022 Jul 1;13:896637. doi: 10.3389/fphar.2022.896637 (PMC9283917; doi:10.3389/fphar.2022.896637)
Supplement: Supplementary file 3 [file DataSheet1.docx]

Supplementary Figure. S1 The chemical structures of coumarins from *A. dahurica*.

Supplementary Figure. S2 The chemical structures of volatile oils from *A. dahurica*.

Supplementary Figure. S3 The chemical structures of alkaloids, phenols, sterols, benzofurans, polyacetylenes and adenosine from *A. dahurica*.
